# Diagnostic and clinical values of non-cardiac ultrasound in COPD: A systematic review

**DOI:** 10.1136/bmjresp-2020-000717

**Published:** 2020-09-25

**Authors:** Jaber S Alqahtani, Tope Oyelade, Jithin Sreedharan, Abdulelah M. Aldhahir, Saeed M Alghamdi, Ahmed M Alrajeh, Abdullah S Alqahtani, Abdullah Alsulayyim, Yousef S Aldabayan, Nowaf Y Alobaidi, Mohammed D. AlAhmari

**Affiliations:** 1 Respiratory Medicine, University College London, London, UK; 2 Prince Sultan Military College of Health Sciences, Dammam, Saudi Arabia; 3 Division of Medicine, University College London, London, UK; 4 Department of Respiratory Care, Faculty of Applied Medical Sciences, Jazan University, Jazan, Saudi Arabia; 5 National Heart and Lung Institute, Imperial College London, London, UK; 6 Faculty of Applied Medical Sciences, Umm Al-Qura University, Makkah, Saudi Arabia; 7 Respiratory Care Department, College of Applied Medical Sciences, King Faisal University, Al-Hasa, Saudi Arabia; 8 Anaesthesia & Critical Care, Division of Clinical Neuroscience, University of Nottingham, Nottingham, UK; 9 Institute of Inflammation and Ageing, University of Birmingham, Birmingham, UK; 10 Respiratory Therapy Department, King Saud bin Abdulaziz University for Health Sciences, Alahsa, Saudi Arabia; 11 Dammam Health Network, Dammam, Saudi Arabia

**Keywords:** COPD exacerbations, COPD pathology, pulmonary rehabilitation

## Abstract

**Background:**

Clinical and research utility of non-cardiac ultrasound (US) in chronic obstructive pulmonary disease (COPD) has been widely investigated. However, there is no systematic review assessing the clinical values of non-cardiac US techniques in COPD.

**Methods:**

We systematically searched electronic databases from inception to 24 June 2020. Two independent reviewers in accordance with the Preferred Reporting Items for Systematic Reviews and Meta-Analyses guidelines extracted data. A narrative synthesis of the results was conducted considering non-cardiac US techniques that looked for diaphragm, muscles and bones in patients with COPD.

**Results:**

In total, 2573 abstracts were screened, and 94 full-text papers were reviewed. A total of 54 studies met the inclusion criteria. Thirty-five studies assessed the diaphragm, while 19 studies evaluated different muscles, including limb muscles and pulmonary lesions in COPD using US. Of the 54 included studies, 30% (16/54) evaluated the changes in either limb muscles or diaphragmatic features before and after physical interventions; 67% (36/54) assessed the correlations between sonographic features and COPD severity. Indeed, 14/15 and 9/13 studies reported a significant reduction in diaphragm excursion and thickness in COPD compared with healthy subjects, respectively; this was correlated significantly with the severity and prognosis of COPD. Three studies reported links between diaphragm length and COPD, where lower diaphragm length correlated with poorer prognosis and outcomes. Quadriceps (rectus femoris), ankle dorsiflexor (tibialis anterior) and vastus lateralis were the most common muscles in COPD assessed by US. More than 70% (12/17) of the studies reported a significant reduction in the cross-sectional area (CSA) of the rectus femoris, rectus femoris and vastus lateralis thickness in COPD compared with healthy subjects. Quadriceps CSA and thickness correlated positively with COPD prognosis, in which patients with reduced quadriceps CSA and thickness have higher risk of exacerbation, readmission and death.

**Conclusion:**

US measurements of diaphragm excursion and thickness, as well as lower limb muscles strength, size and thickness, may provide a safe, portable and effective alternative to radiation-based techniques in diagnosis and prognosis as well as tracking improvement postintervention in patients with COPD.

**Key messages**

- What are the clinical applications and values of non-cardiac ultrasound (US) measurements in chronic obstructive pulmonary disease (COPD)?
- US measurements of diaphragm excursion and thickness, as well as lower limb muscles strength, size and thickness, may provide a safe, portable and effective alternative to radiation-based techniques in diagnosis and prognosis as well as tracking improvement postintervention in patients with COPD.
- This review presents a systematic and in-depth overview of the clinical use of non-cardiac US in COPD and provides evidence that this technique is precise and accurate, serving as a great tool for prognosis and in evaluating response to intervention.

## Introduction

Chronic obstructive pulmonary disease (COPD) is a progressive condition characterised by respiratory symptoms and airflow limitation resulting from airways and lung parenchyma inflammation usually caused by significant exposure to noxious particles and gases.^1^ The WHO estimates that more than three million people die each year from obstructive lung diseases. Globally, COPD is the fourth leading cause of death and it is projected to be the third leading cause of death by 2030.^2^ COPD is commonly associated with inevitable multimorbidities linked with frequent readmissions to hospitals and poor outcomes.^3^ While our understanding of COPD continues to increase, the contributions of comorbidity, such as muscle wasting to clinical outcome, remain a major challenge.

Muscle dysfunction is defined as the loss of muscle strength and endurance.^4^ Functionally, muscle mass and fibre size play a major role in muscle strength. Skeletal muscle dysfunction and mass loss are common systemic symptoms in individuals with COPD.^5^ Moreover, skeletal muscle dysfunction in COPD is linked with morbidity, mortality, poor quality of life and increase hospital admissions.^6^ However, the variable and divergent features of different muscle groups, such as the diaphragm, the lower limbs, abdominal and upper extremity, imply that the mechanism of this dysfunction is non-systemic and need regular monitoring.^7^

A number of imaging technology tools have been used in clinical practice to evaluate skeletal muscle mass; these include ultrasound (US), CT, MRI and spectroscopy.^8^ In this context, we focused only on the US tool and its clinical values. In 1942, US technology was first used in the diagnosis of brain tumour by Dr Karl Dussik.^9^ Furthermore, Ikai and Tetsuo conducted a study by using US technology to measure the cross-sectional area (CSA) of the muscle in living human participants.^10^ Since then, the US technology has been widely used in clinical settings for diagnosing and assessing different conditions, including COPD.^11^ While the use of CT and MRI in COPD population have been explored and described,^12^ there is no dedicated systematic review on the clinical applications of US in COPD and its utility to assess COPD severity and prognosis. Therefore, this review aimed to describe the applications of non-cardiac US and to assess the clinical values in patients with COPD.

## Methods

This systematic review was conducted in accordance with the Preferred Reporting in Systematic Reviews and Meta-Analyses (PRISMA) guidelines. We prospectively registered this review to PROSPERO (registration number CRD42020166924). We searched MEDLINE, Embase and Scopus from inception date to 4 February 2020. The search was updated on 24 June 2020. We used an extensive search strategy developed by a specialised librarian for retrieving this type of evidence, which included the reference list of eligible papers (see online supplemental table S1). All retrieved studies were exported into EndNote to remove duplicates. The remaining studies were exported to Rayyan software. Two independent reviewers performed title, abstracts and full-text screening (table 1).

10.1136/bmjresp-2020-000717.supp1 Supplementary data

**Table 1:** Summary of the outcome measures assessed by US in chronic obstructive pulmonary disease, ordered by the total number of studies

Outcome measures assessed by US	Studies assessed in this outcome, n (references)
Diaphragm muscles	35^13–26 28–32 34–41 43–46 74–76^
Quadriceps (rectus femoris)	10^39 47–55^
Quadriceps (vastus lateralis)	4^53 57–59^
Endobronchial ultrasonography	3^62–64^
Ankle dorsiflexor muscle (tibialis anterior)	2^54 56^
Abdominal muscle	1^61^
Bone mineral density	1^65^
Parasternal intercostal lateral muscle	1^60^

COPD, chronic obstructive pulmonary disease; US, ultrasound.

### Patient and public involvement statement

There was no involvement of patient and public in this paper.

### Inclusion and exclusion criteria

Studies examining patients with COPD using US and all types of studies were included, with no restrictions. US of the cardiac studies were excluded. We excluded studies looking at pulmonary conditions other than COPD; studies where participants have a primary diagnosis of asthma; studies published in any other language other than English; non-full text articles; conference abstracts, editorial reports, correspondence letters, theses and books reviews, or qualitative studies.

### Data collection

Two authors (JSA and TO) independently screened titles and abstracts of potential studies and conflicts were resolved through discussion between the two. Full-text articles of potential studies were then independently read by two authors (JSA and TO) to identify studies meeting the inclusion criteria. The reference lists from all identified studies and reviews were scrutinised for eligible articles. Disagreement on selected papers was resolved through discussion with a third author (AA).

### Quality assessment

Two authors independently evaluated the methodological quality of included cohort studies using a modified version of the Newcastle-Ottawa Scale. Cochrane Risk of Bias Tool was used to assess randomised studies. Any disagreement in the quality assessment was resolved by discussion with a third author.

### Data synthesis

A narrative synthesis of the results was conducted considering US techniques that looked for diaphragm, muscles including lower limb, parasternal intercostal lateral and abdominal muscles and bones in patients with COPD. We could not perform meta-analysis due to the heterogeneity among the studies in terms of design, location and reported measurements.

## Results

**Figure 1:**
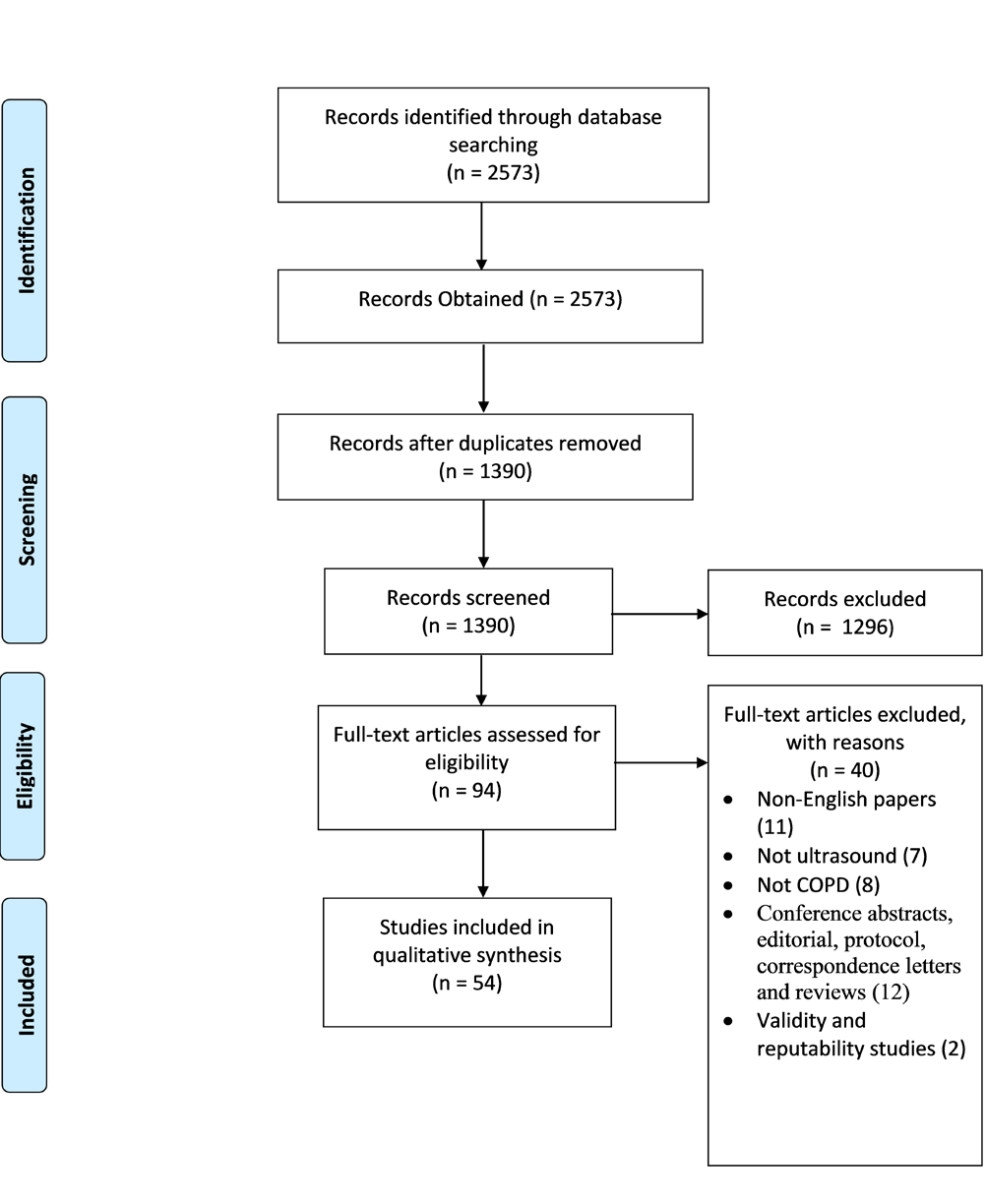
Preferred Reporting Items for Systematic Reviews and Meta-Analyses flowchart.

**Figure 2:**
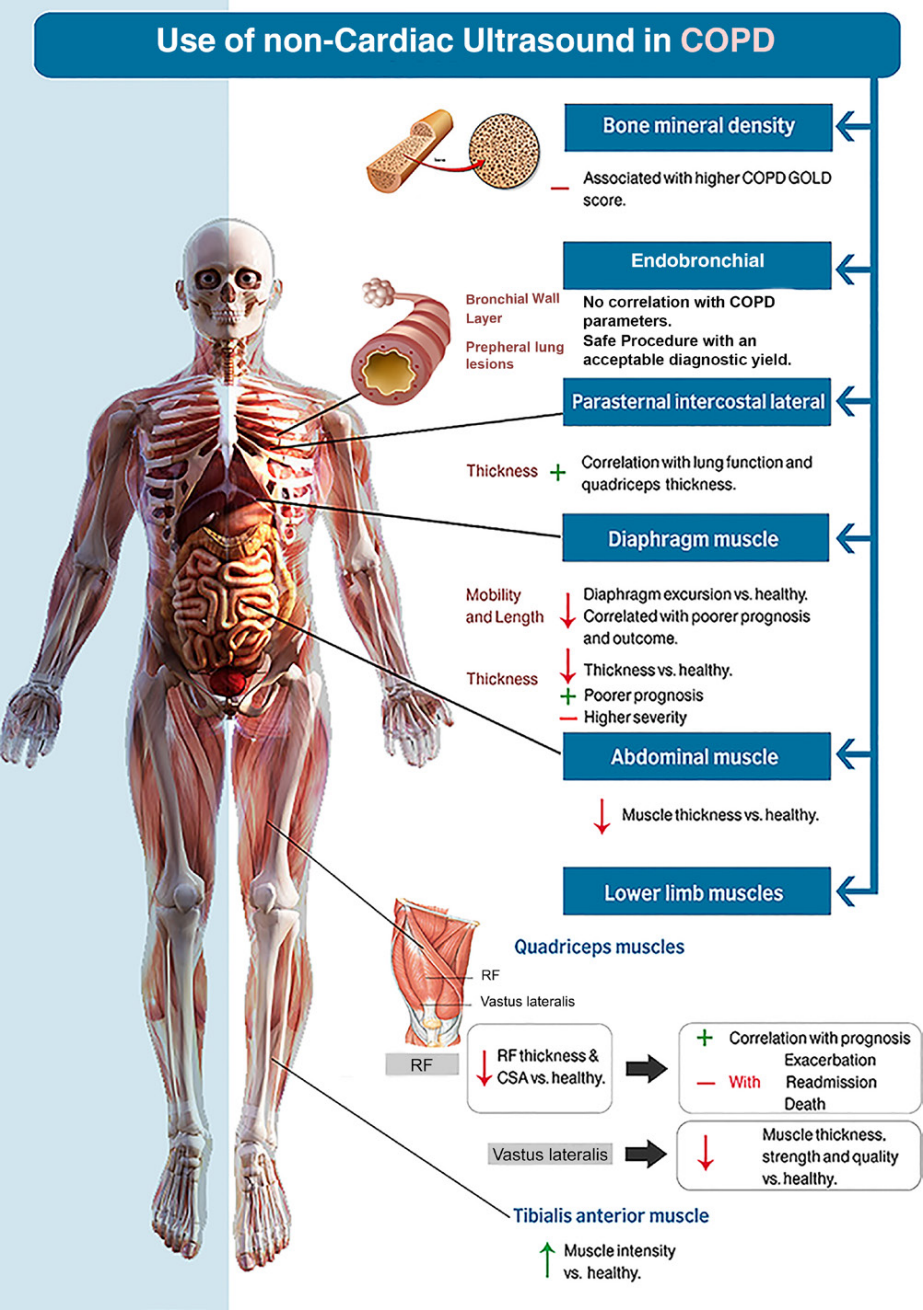
Use of non-cardiac ultrasound in COPD. COPD, chronic obstructive pulmonary disease; CSA, cross-sectional area; GOLD, Global Initiative for Chronic Obstructive Lung Disease; RF, rectus femoris.

An initial search generated 2573 potentially relevant papers, of which 1183 were immediately excluded due to duplication. After the first screening of titles and abstracts, 94 papers were potentially relevant according to the inclusion criteria. An additional 40 papers were excluded after full-text review, which resulted in 54 studies that satisfied all criteria. The reference list of the relevant papers was also examined (figure 1, PRISMA flow diagram). A summary of the included studies is presented in table 1 (for details, see online supplemental tables 1 and 2). Out of 54 studies, 48 were observational and 6 were RCTs. These studies were conducted in 16 countries across Europe, North and South America, Asia and Africa. Thirty-five studies assessed the diaphragm, while 20 studies evaluated skeletal and bronchial smooth muscles in COPD using US. In general, US has been used either to assess the effect of interventions or for diagnosis/prognosis in patients with COPD. Of the 54 included studies, 30% (16/54) evaluated the changes in either skeletal muscle or diaphragmatic features because of physical intervention; the rest assessed the correlations between sonographic features and COPD clinical characteristics. All papers were published between 2000 and 2019 and included 2373 patients with COPD. Of the 48 observational studies, 35% (17/48) had high risk of bias, while 67% (4/6) of the RCTs had high risk of bias in the quality assessment (online supplemental tables 1 and 2). Figure 2 summarises the non-cardiac US use in COPD and the emerging outcomes of use.

10.1136/bmjresp-2020-000717.supp2 Supplementary data

## US and diaphragm

### Diaphragm mobility and length

Of the 35 studies assessing diaphragmatic changes in the COPD, 14 studies^13–26^ reported a significant reduction in diaphragm excursion compared with healthy subjects as assessed by ultrasonography. However, Jain *et al* reported higher diaphragmatic movement in COPD as opposed to other studies.^27^ Five studies involving physical intervention reported significant improvement in diaphragm excursion postintervention,^14 15 28–30^ while three studies reported that diaphragm excursion did not improve following intervention.^31–33^ In addition, three studies reported links between diaphragm length and COPD where lower diaphragm length correlated with poorer prognosis and outcome.^15 34 35^ Overall, clinical intervention targeting diaphragm function such as diaphragmatic breathing training programme and lung volume reduction surgery resulted in significant improvement in lung function.^34 36^

### Diaphragm thickness

Nine of the 35 diaphragm studies reported a significant reduction in US-measured diaphragmatic thickness in patients with COPD compared with healthy subjects.^17 21 27 37–42^ Wasting of diaphragm muscle was correlated with COPD severity and prognosis where a thinner diaphragm was linked with poorer prognosis and higher severity (online supplemental table S2). In contrast, Grosu *et al* reported increased diaphragm thickness in patients with COPD compared with healthy subjects at baseline (0.22±0.07 (cm) vs 0.26±0.06 (cm), p=0.03) with no significant link between rate of diaphragmatic thinning and COPD diagnosis (p=0.36).^43^ Three studies reported no significant difference in diaphragm thickness between patients with COPD and healthy subjects.^44–46^

## US assessment of muscles and bones

### Lower limb muscles

Out of the 14 studies assessing lower limb muscles, the most common muscles of the lower limb that have been assessed with US in COPD are quadriceps (rectus femoris), ankle dorsiflexor (tibialis anterior) and vastus lateralis. Features such as CSA (an assessment of the overall muscle size), echo intensity (assessment of the quality of muscle, whereby high echo intensity equals low muscle quality), thickness and strength have been assessed for diagnosis, prognosis and interventional purposes.

#### Quadriceps (rectus femoris)

Seven studies reported a significant reduction in the CSA of the rectus femoris (RFCSA) in patients with COPD compared with healthy subjects.^39 47–52^ While five studies reported a significant reduction of the rectus femoris thickness in patients with COPD compared with healthy subjects.^13 48 51–54^ In most cases, the CSA and thickness correlated significantly positively with prognosis and negatively with exacerbation, readmission and death of patients with COPD. Two studies reported a significant increase in the muscle echo intensity (poor quality) in COPD, which correlated significantly with severity.^52 53^ Regarding the strength of rectus femoris, while Ramírez-Fuentes *et al* and Maynard-Paquette *et al* reported a significant association, work by Seymour *et al*^54^ reported no link between strength of the quadricep and COPD severity. Moreover, the reduction in the thickness and RFCSA was reported to be significantly improved by physical intervention (exercise training).^55^

#### Ankle dorsiflexor muscle (tibialis anterior)

Three studies assessed tibialis anterior in COPD using ultrasonography. Of these, two studies reported a significant increase in the muscle echo intensity in patients with COPD compared with healthy subjects.^52 56^ Interestingly, they found no significant difference in the CSA of the tibialis anterior between patients with COPD and healthy subjects. In addition, while Maddocks *et al* reported a reduction in strength of the tibialis anterior, another study by Seymour *et al*^54^ found no significant difference between patients with COPD and healthy subjects.

#### Quadriceps (vastus lateralis)

Three studies assessed the features of the vastus lateralis muscle in COPD using US. Two of them reported a significant reduction in vastus lateralis muscle thickness, strength and quality in patients with COPD compared with healthy subjects.^57 58^ However, following exercise training intervention, Alcazar *et al*^59^ reported an increase in the thickness (+11%) and pennation angle/strength (+19%) of the vastus lateralis in patients with COPD.

### Parasternal intercostal lateral and abdominal muscle

Wallbridge *et al* assessed the thickness and echo intensity of parasternal intercostal muscle in patients with COPD using ultrasonography.^60^ Parasternal intercostal muscle thickness was reported to be significantly correlated with lung function (forced expiratory volume in one second (FEV_1_); r=0.33, p<0.05) and quadricep thickness (r=0.32, p<0.05) in patients with COPD. Parasternal intercostal muscle echo intensity was also reported to be correlated with FEV_1_ (r=0.32, p<0.05).

One of the studies included reported a significant reduction in lateral abdominal muscle thickness in COPD compared with healthy control (10.2%±4.4% vs 20.7±5.4%, respectively; p<0.05). Interestingly, thickness was not correlated with lung spirometry (FEV_1_) in patients with COPD and healthy control.^61^

### Bronchial wall features and bone mineral density (BMD)

Górka *et al* measured bronchial wall thickness using endobronchial ultrasonography (EBUS) for the evaluation of emphysema severity in patients with COPD. Bronchial mucosa, submucosa and smooth muscle thickness were not correlated with emphysema score in patients with COPD.^62^ Two studies had assessed peripheral lung lesions in patients with COPD using EBUS and showed that EBUS was safe procedure with an acceptable diagnostic accuracy.^63 64^ In addition, Vrieze *et al* assessed the relationship between BMD and COPD severity using US. Lung spirometry test (FEV_1_) was reported to be a strong predictor of abnormal BMD, where higher COPD GOLD score significantly increased the risk of abnormal BMD.^65^

## Discussion

To the best of our knowledge, this is the first systematic review about the use of non-cardiac US as a diagnostic, prognostic and research tool in patients with COPD. Our systematic review shows that US has been used effectively to understand the relationship between COPD and muscle size, strength and activities. We report here that US is effective in the clinical assessment of the excursion, length and thickness of diaphragm in COPD. We also found that the measurement of skeletal muscle thickness and strength using US provides a precise and accurate clinical assessment of muscle wasting in COPD.

Our review showed that most studies using US to assess the diaphragm reported a reduction in diaphragm excursion in patients with COPD compared with healthy matched subject, and this reduction was correlated significantly with severity and prognosis.^13–26^ The length of the diaphragm was also reported to be shortened in patients with COPD, where US-measured length was positively correlated with prognosis and negatively correlated with severity.^15 34 35^

The result of this review agrees with previous findings where reduced diaphragmatic mobility measured by X-ray was linked with dynamic hyperinflation and air trapping in the lung of patients with COPD compared with healthy subjects.^62^ The dynamic obstruction resulting from the mechanical stretching of the diaphragmatic muscle due to the air trapping and hyperinflation results in a shortened field of movement and reduced diaphragmatic excursion.^66 67^ In addition, following physical intervention, US was able to detect improvement in diaphragm mobility in patients with COPD in most of the included studies.^14 15 28–30^

We also showed that US measurement of diaphragm thickness is another promising technique and has been accurately used to evaluate the clinical characteristics of COPD in many studies. For instance, reduced diaphragm thickness has been significantly correlated with severity and prognosis in patients with COPD.^27^ ^21 37–42^ However, some studies found no significant difference in the thickness of the diaphragmatic muscle between patients with COPD and healthy subjects.^44–46^ Diaphragm muscle atrophy in COPD had been previously described to be mechanistically linked with systemic muscle wasting. This is in the form of chronic loss of type I and type II diaphragm fibres in response to COPD-related physiological changes, such as increased energy expenditure and relative resistance to fatigue.^68 69^

While US has been used in the assessment of skeletal muscles of the lower limbs in some studies, the assessment of the diaphragm using ultrasonography is still the most popular technique (67% vs 35%). In general, thickness, quality, size and strength of the quadriceps, ankle dorsiflexor and vastus lateralis have been studied in COPD using ultrasonography. In this review, most studies reported a significant reduction in CSA of the quadriceps in patients with COPD compared with healthy subjects. These studies also reported that quadriceps CSA and/or thickness correlated positively with prognosis whereby patients with reduced CSA^39 47–52^ and thickness^13 48 51–54^ have a higher risk of exacerbation, readmission and death. The result of this review agrees with previous studies that looked at the risk associated with loss of mid-thigh muscle in patients with COPD. For instance, Marquis *et al* reported an increased risk (50%) of 3-year mortality rate in patients with COPD, with a mid-thigh CSA of <70 cm^2^ compared with those with a CSA of >70 cm^2^.^70^ Thigh muscle wasting in COPD is a well-established clinical presentation of COPD and has been associated with systemic inflammation, chronic hypoxia and lower testosterone level.^71^ Indeed, interventions that limit muscle atrophy or improve muscle regeneration and strength have been shown to improve COPD outcomes and lung function.^55 72^ Such interventions can be tailored to the local healthcare environment in order to reduce exacerbation, hospital readmissions and mortality.^73^

We report here that very few (3/18) studies assessed ankle dorsiflexor muscle using US in patients with COPD. However, of these studies, most reported a reduced muscle quality in the form of increased US echo intensity in patients with COPD compared with healthy subjects.^52 56^ Similarly, the vastus lateralis was assessed using US in only three studies, two of which found a significant reduction in its thickness strength and quality in patients with COPD compared with adults.^57 58^ Interestingly, only one study assessed parasternal intercostal muscle using ultrasonography in COPD and they reported that thickness of parasternal intercostal muscle correlated with lung function and quadriceps thickness.^60^ Thus, US measurement of parasternal muscle thickness is a novel technique that may provide alternative assessment of COPD severity in cases where quadriceps is not assessable.

Furthermore, invasive ultrasonographic technique (EBUS) was applied in three studies. Górka *et al* found no significant relationship between bronchial wall thickness and emphysema score of patients with COPD.^62^ In two other studies, EBUS was used in the assessment of peripheral lung lesion, whereby the technique was found to be safe and have significant diagnostic yield.^63 64^ In another study, Vrieze *et al* reported a strong negative correlation between COPD severity as measured by GOLD (Global Initiative for Chronic Obstructive Lung Disease) score and BMD using US.^65^ Further studies are needed to fully understand the predictive values of these techniques in the context of COPD.

To our knowledge, this review is the first to systematically evaluate existing studies on the use of non-cardiac ultrasonography in patients with COPD. For the first time, we report that the most common uses of non-cardiac US in patients with COPD have been the measurement of diaphragm mobility, length and thickness followed by quadriceps (rectus femoris). In addition, the clinical use of non-cardiac US in COPD is precise and accurate, serving as a great tool for prognosis and in evaluating response to intervention.

This work has several clinical and research implications. First, it highlights the clinical use of US and its effectiveness in COPD—this is particularly important since US provides a safer alternative to X-ray and CT scan, both of which depend on ionising radiation. Also, because of the portability of most US equipment, the point-of-care use of US could provide an assessable, equally efficient method for diagnosis and prognosis in respiratory clinics. The research implications of this review point out the need to have standardised protocols for the use of US in COPD. This could be in the form of global consortium of experts to establish guidelines on the use and interpretation of ultrasonography techniques and measurements. For future researchers, this review provided an overview of the US techniques available and their significance in answering research questions about COPD.

This study has limitations. Heterogeneity exists in study design and reported outcomes, which affects our overall synthesis since different equipment and protocols have been used. We could not perform meta-analysis due to the heterogeneity among the studies in terms of design, location and reported measurements.

In conclusion, US measurements of diaphragm excursion and thickness, as well as lower limb muscles strength, size and thickness, may provide a safe, portable and effective alternative to radiation-based techniques in diagnosis and prognosis, as well as tracking improvement postintervention, in patients with COPD. Future studies are needed to establish the norms of US-based measurements for patients with COPD, other patients with chronic lung disease and healthy subjects.

## References

[1] Rodriguez-Roisin R, Rabe KF, Vestbo J, et al Global initiative for chronic obstructive lung disease (gold) 20th anniversary: a brief history of time. Eur Respir J 2017;50. 10.1183/13993003.00671-2017. [Epub ahead of print: 05 Jul 2017]. 28679615

[2] World Health Organization Chronic obstructive pulmonary disease (COPD), 2017 Available: http://www.who.int/mediacentre/factsheets/fs315/en/

[3] Alqahtani JS, Njoku CM, Bereznicki B, et al Risk factors for all-cause hospital readmission following exacerbation of COPD: a systematic review and meta-analysis. Eur Respir Rev 2020;29:190166. 10.1183/16000617.0166-2019 32499306 PMC9488450

[4] Gosselink R, Troosters T, Decramer M Peripheral muscle weakness contributes to exercise limitation in COPD. Am J Respir Crit Care Med 1996;153:976–80. 10.1164/ajrccm.153.3.8630582 8630582

[5] Jaitovich A, Barreiro E Skeletal muscle dysfunction in chronic obstructive pulmonary disease. what we know and can do for our patients. Am J Respir Crit Care Med 2018;198:175–86. 10.1164/rccm.201710-2140CI 29554438 PMC6058991

[6] Maltais F, Decramer M, Casaburi R, et al An official American thoracic Society/European respiratory Society statement: update on limb muscle dysfunction in chronic obstructive pulmonary disease. Am J Respir Crit Care Med 2014;189:e15–62. 10.1164/rccm.201402-0373ST 24787074 PMC4098112

[7] Polkey MI, Rabe KF Chicken or egg: physical activity in COPD revisited. Eur Respir J 2009;33:227–9. 10.1183/09031936.00176808 19181910

[8] Heymsfield SB, Adamek M, Gonzalez MC, et al Assessing skeletal muscle mass: historical overview and state of the art. J Cachexia Sarcopenia Muscle 2014;5:9–18. 10.1007/s13539-014-0130-5 24532493 PMC3953319

[9] Dussik KT The ultrasonic field as a medical tool. Am J Phys Med 1954;33:5–20. 13124475

[10] Ikai M, Fukunaga T Calculation of muscle strength per unit cross-sectional area of human muscle by means of ultrasonic measurement. Int Z Angew Physiol 1968;26:26–32. 10.1007/BF00696087 5700894

[11] Sorensen B, Hunskaar S Point-of-care ultrasound in primary care: a systematic review of generalist performed point-of-care ultrasound in unselected populations. Ultrasound J 2019;11:31. 10.1186/s13089-019-0145-4 31749019 PMC6868077

[12] Sverzellati N, Molinari F, Pirronti T, et al New insights on COPD imaging via CT and MRI. Int J Chron Obstruct Pulmon Dis 2007;2:301–12. 18229568 PMC2695207

[13] Abbas A, Embarak S, Walaa M, et al Role of diaphragmatic rapid shallow breathing index in predicting weaning outcome in patients with acute exacerbation of COPD. Int J Chron Obstruct Pulmon Dis 2018;13:1655–61. 10.2147/COPD.S161691 29849456 PMC5967374

[14] Corbellini C, Boussuges A, Villafañe JH, et al Diaphragmatic mobility loss in subjects with moderate to very severe COPD may improve after in-patient pulmonary rehabilitation. Respir Care 2018;63:1271–80. 10.4187/respcare.06101 30065081

[15] Crimi C, Heffler E, Augelletti T, et al Utility of ultrasound assessment of diaphragmatic function before and after pulmonary rehabilitation in COPD patients. Int J Chron Obstruct Pulmon Dis 2018;13:3131–9. 10.2147/COPD.S171134 30349221 PMC6183592

[16] Dos Santos Yamaguti WP, Paulin E, Shibao S, et al Air trapping: the major factor limiting diaphragm mobility in chronic obstructive pulmonary disease patients. Respirology 2008;13:138–44. 10.1111/j.1440-1843.2007.01194.x 18197925

[17] Abd El Aziz AA, Elwahsh RA, Abdelaal GA, et al Diaphragmatic assessment in COPD patients by different modalities. Egypt J Chest Dis Tuberc 2017;66:247–50. 10.1016/j.ejcdt.2017.03.006

[18] Evrin T, Korkut S, Ozturk Sonmez L, et al Evaluating stable chronic obstructive pulmonary disease by ultrasound. Emerg Med Int 2019;2019:1–8. 10.1155/2019/5361620 PMC6766158 31637058

[19] He L, Zhang W, Zhang J, et al Diaphragmatic motion studied by M-mode ultrasonography in combined pulmonary fibrosis and emphysema. Lung 2014;192:553–61. 10.1007/s00408-014-9594-5 24818955

[20] Kang HW, Kim TO, Lee BR, et al Influence of diaphragmatic mobility on hypercapnia in patients with chronic obstructive pulmonary disease. J Korean Med Sci 2011;26:1209–13. 10.3346/jkms.2011.26.9.1209 21935278 PMC3172660

[21] Lim SY, Lim G, Lee YJ, et al Ultrasound assessment of diaphragmatic function during acute exacerbation of chronic obstructive pulmonary disease: a pilot study. Int J Chron Obstruct Pulmon Dis 2019;14:2479–84. 10.2147/COPD.S214716 31806957 PMC6844220

[22] Paulin E, Yamaguti WPS, Chammas MC, et al Influence of diaphragmatic mobility on exercise tolerance and dyspnea in patients with COPD. Respir Med 2007;101:2113–8. 10.1016/j.rmed.2007.05.024 17644365

[23] Scheibe N, Sosnowski N, Pinkhasik A, et al Sonographic evaluation of diaphragmatic dysfunction in COPD patients. Int J Chron Obstruct Pulmon Dis 2015;10:10. 10.2147/COPD.S85659 PMC4574853 26392767

[24] Sun Q, Liu L, Pan C, et al Effects of neurally adjusted ventilatory assist on air distribution and dead space in patients with acute exacerbation of chronic obstructive pulmonary disease. Crit Care 2017;21:126. 10.1186/s13054-017-1714-1 28578708 PMC5455203

[25] Zhang X, Yuan J, Zhan Y, et al Evaluation of diaphragm ultrasound in predicting extubation outcome in mechanically ventilated patients with COPD. Ir J Med Sci 2020;189:661–8. 10.1007/s11845-019-02117-1 31691888 PMC7223179

[26] Souza RMP, Cardim AB, Maia TO, et al Inspiratory muscle strength, diaphragmatic mobility, and body composition in chronic obstructive pulmonary disease. Physiother Res Int 2019;24:e1766. 10.1002/pri.1766 30628141

[27] Jain S, Nair G, Nuchin A, et al Study of the diaphragm in chronic obstructive pulmonary disease using ultrasonography. Lung India 2019;36:299–303. 10.4103/lungindia.lungindia_466_18 31290414 PMC6625233

[28] Bhatt SP, Luqman-Arafath TK, Gupta AK, et al Volitional pursed lips breathing in patients with stable chronic obstructive pulmonary disease improves exercise capacity. Chron Respir Dis 2013;10:5–10. 10.1177/1479972312464244 23149383

[29] Priori R, Aliverti A, Albuquerque AL, et al The effect of posture on asynchronous chest wall movement in COPD. J Appl Physiol 2013;114:1066–75. 10.1152/japplphysiol.00414.2012 23412901

[30] Rocha T, Souza H, Brandão DC, et al The manual diaphragm release technique improves diaphragmatic mobility, inspiratory capacity and exercise capacity in people with chronic obstructive pulmonary disease: a randomised trial. J Physiother 2015;61:182–9. 10.1016/j.jphys.2015.08.009 26386894

[31] Andrews SM, Deoghare HV, Mills PK, et al Pulmonary rehabilitation maintenance program may prevent accelerated FEV1 decline in patients with COPD. Clin Pulm Med 2017;24:143–8. 10.1097/CPM.0000000000000213

[32] Nair A, Alaparthi GK, Krishnan S, et al Comparison of diaphragmatic stretch technique and manual diaphragm release technique on diaphragmatic excursion in chronic obstructive pulmonary disease: a randomized crossover trial. Pulm Med 2019;2019:1–7. 10.1155/2019/6364376 PMC6335861 30719351

[33] Bhatt SP, Guleria R, Luqman-Arafath TK, et al Effect of TRIPOD position on objective parameters of respiratory function in stable chronic obstructive pulmonary disease. Indian J Chest Dis Allied Sci 2009;51:83–5. 19445443

[34] Gorman RB, McKenzie DK, Butler JE, et al Diaphragm length and neural drive after lung volume reduction surgery. Am J Respir Crit Care Med 2005;172:1259–66. 10.1164/rccm.200412-1695OC 16109977

[35] Gorman RB, McKenzie DK, Pride NB, et al Diaphragm length during tidal breathing in patients with chronic obstructive pulmonary disease. Am J Respir Crit Care Med 2002;166:1461–9. 10.1164/rccm.200111-087OC 12406839

[36] Yamaguti WP, Claudino RC, Neto AP, et al Diaphragmatic breathing training program improves abdominal motion during natural breathing in patients with chronic obstructive pulmonary disease: a randomized controlled trial. Arch Phys Med Rehabil 2012;93:571–7. 10.1016/j.apmr.2011.11.026 22464088

[37] Elsawy SB Impact of chronic obstructive pulmonary disease severity on diaphragm muscle thickness. Egypt J Chest Dis Tuberc 2017;66:587–92. 10.1016/j.ejcdt.2017.08.002

[38] Marchioni A, Castaniere I, Tonelli R, et al Ultrasound-assessed diaphragmatic impairment is a predictor of outcomes in patients with acute exacerbation of chronic obstructive pulmonary disease undergoing noninvasive ventilation. Crit Care 2018;22:109. 10.1186/s13054-018-2033-x 29703214 PMC5921560

[39] Maynard-Paquette A-C, Poirier C, Chartrand-Lefebvre C, et al Ultrasound evaluation of the quadriceps muscle contractile index in patients with stable chronic obstructive pulmonary disease: relationships with clinical symptoms, disease severity and diaphragm contractility. Int J Chron Obstruct Pulmon Dis 2020;15:79–88. 10.2147/COPD.S222945 32021146 PMC6957010

[40] Okura K, Kawagoshi A, Iwakura M, et al Contractile capability of the diaphragm assessed by ultrasonography predicts nocturnal oxygen saturation in COPD. Respirology 2017;22:301–6. 10.1111/resp.12897 27611719

[41] Smargiassi A, Inchingolo R, Tagliaboschi L, et al Ultrasonographic assessment of the diaphragm in chronic obstructive pulmonary disease patients: relationships with pulmonary function and the influence of body composition - a pilot study. Respiration 2014;87:364–71. 10.1159/000358564 24732295

[42] Cimsit C, Bekir M, Karakurt S, et al Ultrasound assessment of diaphragm thickness in COPD. MMJ 2016;29:8–13. 10.5472/MMJoa.2901.02

[43] Grosu HB, Ost DE, Lee YI, et al Diaphragm muscle thinning in subjects receiving mechanical ventilation and its effect on extubation. Respir Care 2017;62:904–11. 10.4187/respcare.05370 28351903 PMC6373860

[44] Baria MR, Shahgholi L, Sorenson EJ, et al B-mode ultrasound assessment of diaphragm structure and function in patients with COPD. Chest 2014;146:680–5. 10.1378/chest.13-2306 24700122 PMC4151360

[45] Eryüksel E, Cimşit C, Bekir M, et al Diaphragmatic thickness fraction in subjects at high-risk for COPD exacerbations. Respir Care 2017;62:1565–70. 10.4187/respcare.05646 28874613

[46] Ogan N, Aydemir Y, EVrin T, et al Diaphragmatic thickness in chronic obstructive lung disease and relationship with clinical severity parameters. Turk J Med Sci 2019;49:1073–8. 10.3906/sag-1901-164 31293145 PMC7018351

[47] Greening NJ, Harvey-Dunstan TC, Chaplin EJ, et al Bedside assessment of quadriceps muscle by ultrasound after admission for acute exacerbations of chronic respiratory disease. Am J Respir Crit Care Med 2015;192:810–6. 10.1164/rccm.201503-0535OC 26068143 PMC4613897

[48] Nijholt W, Beek LT, Hobbelen JSM, et al The added value of ultrasound muscle measurements in patients with COPD: an exploratory study. Clin Nutr ESPEN 2019;30:152–8. 10.1016/j.clnesp.2019.01.001 30904216

[49] Ramírez-Fuentes C, Mínguez-Blasco P, Ostiz F, et al Ultrasound assessment of rectus femoris muscle in rehabilitation patients with chronic obstructive pulmonary disease screened for sarcopenia: correlation of muscle size with quadriceps strength and fat-free mass. Eur Geriatr Med 2019;10:89–97. 10.1007/s41999-018-0130-7 32720275

[50] Seymour JM, Ward K, Sidhu PS, et al Ultrasound measurement of rectus femoris cross-sectional area and the relationship with quadriceps strength in COPD. Thorax 2009;64:418–23. 10.1136/thx.2008.103986 19158125

[51] Shrikrishna D, Patel M, Tanner RJ, et al Quadriceps wasting and physical inactivity in patients with COPD. Eur Respir J 2012;40:1115–22. 10.1183/09031936.00170111 22362854

[52] Ye X, Wang M, Xiao H Echo intensity of the rectus femoris in stable COPD patients. Int J Chron Obstruct Pulmon Dis 2017;12:3007–15. 10.2147/COPD.S143645 29075109 PMC5648322

[53] Cruz-Montecinos C, Guajardo-Rojas C, Montt E, et al Sonographic measurement of the quadriceps muscle in patients with chronic obstructive pulmonary disease: functional and clinical implications. J Ultrasound Med 2016;35:2405–12. 10.7863/ultra.15.11032 27698182

[54] Seymour JM, Ward K, Raffique A, et al Quadriceps and ankle dorsiflexor strength in chronic obstructive pulmonary disease. Muscle Nerve 2012;46:548–54. 10.1002/mus.23353 22987696

[55] Menon MK, Houchen L, Harrison S, et al Ultrasound assessment of lower limb muscle mass in response to resistance training in COPD. Respir Res 2012;13:119. 10.1186/1465-9921-13-119 23273255 PMC3560243

[56] Maddocks M, Jones M, Snell T, et al Ankle dorsiflexor muscle size, composition and force with ageing and chronic obstructive pulmonary disease. Exp Physiol 2014;99:1078–88. 10.1113/expphysiol.2014.080093 24928952

[57] Coratella G, Rinaldo N, Schena F Quadriceps concentric-eccentric force and muscle architecture in COPD patients vs healthy men. Hum Mov Sci 2018;59:88–95. 10.1016/j.humov.2018.03.015 29625361

[58] Navarro-Cruz R, Alcazar J, Rodriguez-Lopez C, et al The effect of the Stretch-Shortening cycle in the force-velocity relationship and its association with physical function in older adults with COPD. Front Physiol 2019;10:316. 10.3389/fphys.2019.00316 30971950 PMC6443992

[59] Alcazar J, Losa-Reyna J, Rodriguez-Lopez C, et al Effects of concurrent exercise training on muscle dysfunction and systemic oxidative stress in older people with COPD. Scand J Med Sci Sports 2019;29:1591–603. 10.1111/sms.13494 31169924

[60] Wallbridge P, Parry SM, Das S, et al Parasternal intercostal muscle ultrasound in chronic obstructive pulmonary disease correlates with spirometric severity. Sci Rep 2018;8:1–9. 10.1038/s41598-018-33666-7 30323179 PMC6189142

[61] Kaneko H, Maruyama H, Sato H Relationship between expiratory activity of the lateral abdominal muscle and exercise tolerance in chronic obstructive pulmonary disease. J Phys Ther Sci 2008;20:147–51. 10.1589/jpts.20.147

[62] Górka K, Soja J, Jakieła B, et al Relationship between the thickness of bronchial wall layers, emphysema score, and markers of remodeling in bronchoalveolar lavage fluid in patients with chronic obstructive pulmonary disease. Pol Arch Med Wewn 2016;126:402–10. 10.20452/pamw.3461 27362393

[63] Georgiou HD, Taverner J, Irving LB, et al Safety and efficacy of radial EBUS for the investigation of peripheral pulmonary lesions in patients with advanced COPD. J Bronchology Interv Pulmonol 2016;23:192–8. 10.1097/LBR.0000000000000288 27454473

[64] Lee KM, Lee G, Kim A, et al Clinical outcomes of radial probe endobronchial ultrasound using a guide sheath for diagnosis of peripheral lung lesions in patients with pulmonary emphysema. Respir Res 2019;20:177. 10.1186/s12931-019-1149-0 31387600 PMC6683511

[65] Vrieze A, de Greef MHG, Wijkstra PJ, et al Low bone mineral density in COPD patients related to worse lung function, low weight and decreased fat-free mass. Osteoporos Int 2007;18:1197–202. 10.1007/s00198-007-0355-7 17347789

[66] Rocha FR, Brüggemann AKV, Francisco DdeS, et al Diaphragmatic mobility: relationship with lung function, respiratory muscle strength, dyspnea, and physical activity in daily life in patients with COPD. J Bras Pneumol 2017;43:32–7. 10.1590/s1806-37562016000000097 28380186 PMC5790674

[67] Reid WD, Samrai B Respiratory muscle training for patients with chronic obstructive pulmonary disease. Phys Ther 1995;75:996–1005. 10.1093/ptj/75.11.996 7480129

[68] Levine S, Kaiser L, Leferovich J, et al Cellular adaptations in the diaphragm in chronic obstructive pulmonary disease. N Engl J Med 1997;337:1799–806. 10.1056/NEJM199712183372503 9400036

[69] Ottenheijm CAC, Heunks LMA, Dekhuijzen RPN Diaphragm adaptations in patients with COPD. Respir Res 2008;9:12. 10.1186/1465-9921-9-12 18218129 PMC2248576

[70] Marquis K, Debigaré R, Lacasse Y, et al Midthigh muscle cross-sectional area is a better predictor of mortality than body mass index in patients with chronic obstructive pulmonary disease. Am J Respir Crit Care Med 2002;166:809–13. 10.1164/rccm.2107031 12231489

[71] Wüst RCI, Degens H Factors contributing to muscle wasting and dysfunction in COPD patients. Int J Chron Obstruct Pulmon Dis 2007;2:289–300. 18229567 PMC2695204

[72] Aldhahir AM, Rajeh AMA, Aldabayan YS, et al Nutritional supplementation during pulmonary rehabilitation in COPD: a systematic review. Chron Respir Dis 2020;17:147997312090495–53. 10.1177/1479973120904953 PMC7019390 32054293

[73] Njoku CM, Alqahtani JS, Wimmer BC, et al Risk factors and associated outcomes of hospital readmission in COPD: a systematic review. Respir Med 2020:105988 10.1016/j.rmed.2020.105988 33190738

[74] Bhatt SP, Guleria R, Luqman-Arafath TK, et al Effect of TRIPOD position on objective parameters of respiratory function in stable chronic obstructive pulmonary disease. Indian J Chest Dis Allied Sci 2009;51:83. 19445443

[75] Cimsit C, Bekir M, Karakurt S, et al Ultrasound assessment of diaphragm thickness in COPD. MMJ 2016;29:8 10.5472/MMJoa.2901.02

[76] McKenzie DK, Gorman RB, Tolman J, et al Estimation of diaphragm length in patients with severe chronic obstructive pulmonary disease. Respir Physiol 2000;123:225–34. 10.1016/S0034-5687(00)00172-9 11007989

