# Supplementary material for: Diagnostic and clinical values of non-cardiac ultrasound in COPD: A systematic review
Source: BMJ Open Respir Res. 2020 Sep 25;7(1):e000717. doi: 10.1136/bmjresp-2020-000717 (PMC7520906; doi:10.1136/bmjresp-2020-000717)
Supplement: Supplementary data [file bmjresp-2020-000717supp001.pdf]

Table S1. Search strategy

_Database: Ovid MEDLINE(R) and Epub Ahead of Print, In-Process & Other Non-Indexed Citations and Daily <1946 to February 04, 2020>

Search Strategy:

----

1 lung diseases, obstructive/ or pulmonary disease, chronic obstructive/

2 chronic airflow obstruction.mp. [mp=title, abstract, original title, name of substance word, subject heading word, floating sub-heading word, keyword heading word, organism supplementary concept word, protocol supplementary concept word, rare disease supplementary concept word, unique identifier, synonyms]

3 copd.mp. [mp=title, abstract, original title, name of substance word, subject heading word, floating sub-heading word, keyword heading word, organism supplementary concept word, protocol supplementary concept word, rare disease supplementary concept word, unique identifier, synonyms]

4 chronic obstructive airway disease.mp. [mp=title, abstract, original title, name of substance word, subject heading word, floating sub-heading word, keyword heading word, organism supplementary concept word, protocol supplementary concept word, rare disease supplementary concept word, unique identifier, synonyms]

5 chronic obstructive lung disease.mp. [mp=title, abstract, original title, name of substance word, subject heading word, floating sub-heading word, keyword heading word, organism supplementary concept word, protocol supplementary concept word, rare disease supplementary concept word, unique identifier, synonyms]

6 chronic obstructive pulmonary disease.mp. [mp=title, abstract, original title, name of substance word, subject heading word, floating sub-heading word, keyword heading word, organism supplementary concept word, protocol supplementary concept word, rare disease supplementary concept word, unique identifier, synonyms]

7 Ultrasonography/

8 (ultrasound or ultrason* or tomograph* or imag*).mp. [mp=title, abstract, original title, name of substance word, subject heading word, floating sub-heading word, keyword heading word, organism supplementary concept word, protocol supplementary concept word, rare disease supplementary concept word, unique identifier, synonyms]

9 lung ultrasound.mp. [mp=title, abstract, original title, name of substance word, subject heading word, floating sub-heading word, keyword heading word, organism supplementary concept word, protocol supplementary concept word, rare disease supplementary concept word, unique identifier, synonyms]

10 echograph*.mp. [mp=title, abstract, original title, name of substance word, subject heading word, floating sub-heading word, keyword heading word, organism supplementary concept word, protocol supplementary concept word, rare disease supplementary concept word, unique identifier, synonyms]

11 echotomograph*.mp. [mp=title, abstract, original title, name of substance word, subject heading word, floating sub-heading word, keyword heading word, organism supplementary concept word, protocol supplementary concept word, rare disease supplementary concept word, unique identifier, synonyms]

12 medical sonograph*.mp. [mp=title, abstract, original title, name of substance word, subject heading word, floating sub-heading word, keyword heading word, organism supplementary concept word, protocol supplementary concept word, rare disease supplementary concept word, unique identifier, synonyms]

13 exp Muscles/

14 muscle*.mp. [mp=title, abstract, original title, name of substance word, subject heading word, floating sub-heading word, keyword heading word, organism supplementary concept word, protocol supplementary concept word, rare disease supplementary concept word, unique identifier, synonyms]

15 exp Diaphragm/

16 diaphragm*.mp. [mp=title, abstract, original title, name of substance word, subject heading word, floating sub-heading word, keyword heading word, organism supplementary concept word, protocol supplementary concept word, rare disease supplementary concept word, unique identifier, synonyms]

17 7 or 8 or 9 or 10 or 11 or 12

18 13 or 14 or 15 or 16

19 1 or 2 or 3 or 4 or 5 or 6

20 17 and 18 and 19

****

Table S2. Details of the quality of Cohort studies

First author	Population representative	Sample size adequate	Confounders	Statistical analysis	Missing data	Methodology of the outcome	Objective assessment	OVERALL (0-3, higher score = lower risk of bias)
Abbas et.al 2020 [1]	0	0	0	3	0	2	1	0.85
Baria et al 2014 [2]	3	2	3	1	3	3	3	2.6
Bhatt et al. 2009 [3]	0	0	1	1	2	1	2	1
Cimşit et al. 2016 [4]	3	1	3	3	3	3	3	2.7
Coratella et.al. 2018 [5]	1	2	0	0	1	1	1	0.85
Corbellini et al. 2018 [6]	3	1	3	2	3	3	2	2.4
Crimi et.al. 2018 [7]	1	1	3	3	3	3	1	2.1
Cruz-Montecinos et al.2016 [8]	0	2	2	2	2	2	1	1.6
El Aziz, Amal et al. 2017 [9]	3	1	3	2	3	3	3	2.6
Elsawy et al. 2017 [10]	1	2	1	2	3	2	2	1.9
Eryüksel et al. 2017 [11]	0	1	1	2	1	2	2	1.3
Evrin et al. 2019 [12]	2	2	1	2	1	2	2	1.7
Georgiou et al. 2016 [13]	2	1	1	2	2	2	2	1.7
Górka et al 2016 [14]	0	0	1	2	1	1	2	1
Gorman et al. 2005 [15]	2	0	3	3	3	3	3	2.4
Gorman et al. et al 2002 [16]	2	0	3	3	3	3	3	2.4
Greening et al 2015 [17]	3	3	2	3	3	3	2	2.7
Grosu et al. 2017 [18]	2	1	2	2	3	2	3	2.1
He L et al. 2014 [19]	1	1	1	2	2	2	2	1.6
J M Seymour et al. 2009 [20]	3	2	2	3	3	3	3	2.7
Jain et al. 2019 [21]	3	1	1	2	3	1	3	2

Kaneko et.al 2008 [22]	0	1	0	0	1	1	1	0.6
Kang et al. 2011 [23]	0	0	1	1	2	2	2	1.1
Lee KM et al. 2019 [24]	1	1	1	2	1	2	2	1.4
Lim et al. 2019 [25]	1	0	0	1	2	2	1	1
Maddocks et al. 2014 [26]	3	3	1	3	3	3	3	2.7
Marchioni et.al 2018 [27]	3	2	2	3	3	3	1	2.4
Maynard-Paquette et al. 2020 [28]	3	2	2	3	2	3	2	2.4
McKenzie et al. 2000 [29]	2	0	2	3	3	3	3	2.2
Menon et al. 2012 [30]	3	2	3	3	3	3	3	2.8
Navarro-Cruz et.al. 2019 [31]	3	1	2	3	3	3	2	2.4
Nijholt et al. 2019 [32]	3	1	2	3	3	3	3	2.5
Ogan et al. 2019 [33]	0	1	1	2	0	2	2	1.1
Okura et al. 2017 [34]	2	2	3	2	3	3	3	2.6
Paulin et al. 2007 [35]	1	1	1	2	2	2	2	1.6
Priori et al. 2013 [36]	0	1	1	1	3	1	2	1.1
Ramírez-Fuentes et al. 2019 [37]	2	2	3	3	2	3	3	2.6
Scheibe et al. 2015 [38]	1	0	1	1	2	2	2	1.2
Seymour et.al 2012 [39]	1	0	0	2	0	1	2	0.85
Shrikrishna et al. 2012 [40]	3	2	2	3	1	3	2	2.1
Smargiassi et al. 2014 [41]	3	2	2	3	3	3	2	2.5
Souza et.al. 2017 [42]	1	0	0	1	0	1	2	0.7
Sun et al. 2017 [43]	0	1	1	1	2	1	1	1
Vrieze et.al. 2007 [44]	1	2	1	2	2	2	2	1.7
Wallbridge et.al 2018 [45]	1	1	1	2	2	2	2	1.6

Yamaguti et al 2008 [46]	3	0	2	3	3	2	3	2.3
Ye X et al. 2017 [47]	1	1	2	0	2	1	0	1
Zhang et al. 2019 [48]	0	0	1	2	0	2	2	1

0 = definitely no (high risk of bias); 1 = mostly no; 2 = Mostly yes; 3 = definitely yes (low risk of bias)

Table S3. Risk of bias of the included RCT

First author	Random Sequence generation	Allocation concealment	Selective reporting	Blinding subject+ personnel	Blinding outcome assessment	incomplete outcome data)	Other source of bias	OVERALL (0-7, higher score = higher risk of bias)
Bhatt et.al. 2013 [49]	High	High	Low	High	High	Low	Unclear	5
Andrews et al. 2017 [50]	Unclear	Low	High	High	low	Unclear	High	5
Alcazar et al. 2019 [51]	unclear	unclear	unclear	unclear	unclear	low	low	5
Rocha et.al. 2015 [52]	low	low	low	low	low	low	low	7
Nair et al.2019 [53]	Low	Low	Low	High	High	Low	High	3
Yamaguti WP et al. 2012 [54]	Low	High	Low	High	Low	Low	Low	2

References:

1. Abbas A, Embarak S, Walaa M, et al. Role of diaphragmatic rapid shallow breathing index in predicting weaning outcome in patients with acute exacerbation of COPD. *International journal of chronic obstructive pulmonary disease* 2018;13:1655.
2. Baria MR, Shahgholi L, Sorenson EJ, et al. B-mode ultrasound assessment of diaphragm structure and function in patients with COPD. *Chest* 2014;146(3):680-85.
3. Bhatt S, Guleria R, Luqman-Arafath T, et al. Effect of tripod position on objective parameters of respiratory function in stable chronic obstructive pulmonary disease. *The Indian journal of chest diseases & allied sciences* 2009;51(2):83.
4. Cimsit C, Bekir M, Karakurt S, et al. Ultrasound assessment of diaphragm thickness in COPD. 2016
5. Coratella G, Rinaldo N, Schena F. Quadriceps concentric-eccentric force and muscle architecture in COPD patients vs healthy men. *Human movement science* 2018;59:88-95.
6. Corbellini C, Boussuges A, Villafañe JH, et al. Diaphragmatic mobility loss in subjects with moderate to very severe COPD may improve after in-patient pulmonary rehabilitation. *Respiratory care* 2018;63(10):1271-80.
7. Crimi C, Heffler E, Augelletti T, et al. Utility of ultrasound assessment of diaphragmatic function before and after pulmonary rehabilitation in COPD patients. *International Journal of Chronic Obstructive Pulmonary Disease* 2018;13:3131.
8. Cruz-Montecinos C, Guajardo-Rojas C, Montt E, et al. Sonographic measurement of the quadriceps muscle in patients with chronic obstructive pulmonary disease: functional and clinical implications. *Journal of Ultrasound in Medicine* 2016;35(11):2405-12.
9. El Aziz AAA, Elwahsh RA, Abdelaal GA, et al. Diaphragmatic assessment in COPD patients by different modalities. *Egyptian journal of chest diseases and tuberculosis* 2017;66(2):247-50.
10. Elsawy SB. Impact of chronic obstructive pulmonary disease severity on diaphragm muscle thickness. *Egyptian Journal of Chest Diseases and Tuberculosis* 2017;66(4):587-92.
11. Eryüksel E, Cimşit C, Bekir M, et al. Diaphragmatic thickness fraction in subjects at high-risk for COPD exacerbations. *Respiratory care* 2017;62(12):1565-70.
12. Evrin T, Korkut S, Ozturk Sonmez L, et al. Evaluating Stable Chronic Obstructive Pulmonary Disease by Ultrasound. *Emergency medicine international* 2019;2019
13. Georgiou HD, Taverner J, Irving LB, et al. Safety and efficacy of radial EBUS for the investigation of peripheral pulmonary lesions in patients with advanced COPD. *J Bronchology Interv Pulmonol* 2016;23(3):192-98.
14. Górka K, Soja J, Jakieła B, et al. Relationship between the thickness of bronchial wall layers, emphysema score, and markers of remodeling in bronchoalveolar lavage fluid in patients with chronic obstructive pulmonary disease. *Pol Arch Med Wewn* 2016;126(6):402-10.
15. Gorman RB, McKenzie DK, Butler JE, et al. Diaphragm length and neural drive after lung volume reduction surgery. *Am J Respir Crit Care Med* 2005;172(10):1259-66. doi: 10.1164/rccm.200412-1695OC [published Online First: 2005/08/20]
16. Gorman RB, McKenzie DK, Pride NB, et al. Diaphragm length during tidal breathing in patients with chronic obstructive pulmonary disease. *Am J Respir Crit Care Med* 2002;166(11):1461-9. doi: 10.1164/rccm.200111-087OC [published Online First: 2002/10/31]
17. Greening NJ, Harvey-Dunstan TC, Chaplin EJ, et al. Bedside assessment of quadriceps muscle by ultrasound after admission for acute exacerbations of chronic respiratory disease. *American journal of respiratory and critical care medicine* 2015;192(7):810-16.

18. Grosu HB, Ost DE, Lee YI, et al. Diaphragm Muscle Thinning in Subjects Receiving Mechanical Ventilation and Its Effect on Extubation. *Respir Care* 2017;62(7):904-11. doi: 10.4187/respcare.05370 [published Online First: 2017/03/30]
19. He L, Zhang W, Zhang J, et al. Diaphragmatic motion studied by M-mode ultrasonography in combined pulmonary fibrosis and emphysema. *Lung* 2014;192(4):553-61.
20. Seymour JM, Ward K, Sidhu PS, et al. Ultrasound measurement of rectus femoris cross-sectional area and the relationship with quadriceps strength in COPD. *Thorax* 2009;64(5):418-23.
21. Jain S, Nair G, Nuchin A, et al. Study of the diaphragm in chronic obstructive pulmonary disease using ultrasonography. *Lung India: Official Organ of Indian Chest Society* 2019;36(4):299.
22. Kaneko H, Maruyama H, Sato H. Relationship between expiratory activity of the lateral abdominal muscle and exercise tolerance in chronic obstructive pulmonary disease. *Journal of Physical Therapy Science* 2008;20(2):147-51.
23. Kang HW, Kim TO, Lee BR, et al. Influence of diaphragmatic mobility on hypercapnia in patients with chronic obstructive pulmonary disease. *Journal of Korean medical science* 2011;26(9):1209-13.
24. Lee KM, Lee G, Kim A, et al. Clinical outcomes of radial probe endobronchial ultrasound using a guide sheath for diagnosis of peripheral lung lesions in patients with pulmonary emphysema. *Respiratory research* 2019;20(1):177.
25. Lim SY, Lim G, Lee YJ, et al. Ultrasound Assessment Of Diaphragmatic Function During Acute Exacerbation Of Chronic Obstructive Pulmonary Disease: A Pilot Study. *International Journal of Chronic Obstructive Pulmonary Disease* 2019;14:2479.
26. Maddocks M, Jones M, Snell T, et al. Ankle dorsiflexor muscle size, composition and force with ageing and chronic obstructive pulmonary disease. *Exp Physiol* 2014;99(8):1078-88. doi: 10.1113/expphysiol.2014.080093 [published Online First: 2014/06/15]
27. Marchioni A, Castaniere I, Tonelli R, et al. Ultrasound-assessed diaphragmatic impairment is a predictor of outcomes in patients with acute exacerbation of chronic obstructive pulmonary disease undergoing noninvasive ventilation. *Critical Care* 2018;22(1):109.
28. Maynard-Paquette A-C, Poirier C, Chartrand-Lefebvre C, et al. Ultrasound Evaluation of the Quadriceps Muscle Contractile Index in Patients with Stable Chronic Obstructive Pulmonary Disease: Relationships with Clinical Symptoms, Disease Severity and Diaphragm Contractility. *International Journal of Chronic Obstructive Pulmonary Disease* 2020;15:79.
29. McKenzie DK, Gorman RB, Tolman J, et al. Estimation of diaphragm length in patients with severe chronic obstructive pulmonary disease. *Respiration physiology* 2000;123(3):225-34.
30. Menon MK, Houchen L, Harrison S, et al. Ultrasound assessment of lower limb muscle mass in response to resistance training in COPD. *Respiratory research* 2012;13(1):119.
31. Navarro-Cruz R, Alcazar J, Rodriguez-Lopez C, et al. The effect of the stretch-shortening cycle in the force-velocity relationship and its association with physical function in older adults with COPD. *Frontiers in physiology* 2019;10:316.
32. Nijholt W, ter Beek L, Hobbelen JS, et al. The added value of ultrasound muscle measurements in patients with COPD: An exploratory study. *Clinical nutrition ESPEN* 2019;30:152-58.
33. Ogan N, Aydemir Y, Evrin T, et al. Diaphragmatic thickness in chronic obstructive lung disease and relationship with clinical severity parameters. *Turkish journal of medical sciences* 2019;49(4):1073-78.

34. Okura K, Kawagoshi A, Iwakura M, et al. Contractile capability of the diaphragm assessed by ultrasonography predicts nocturnal oxygen saturation in COPD. *Respirology*;22(2):301-06.
35. Paulin E, Yamaguti W, Chammas M, et al. Influence of diaphragmatic mobility on exercise tolerance and dyspnea in patients with COPD. *Respiratory medicine* 2007;101(10):2113-18.
36. Priori R, Aliverti A, Albuquerque AL, et al. The effect of posture on asynchronous chest wall movement in COPD. *Journal of Applied Physiology* 2013;114(8):1066-75.
37. Ramírez-Fuentes C, Mínguez-Blasco P, Ostiz F, et al. Ultrasound assessment of rectus femoris muscle in rehabilitation patients with chronic obstructive pulmonary disease screened for sarcopenia: correlation of muscle size with quadriceps strength and fat-free mass. *European Geriatric Medicine* 2019;10(1):89-97.
38. Scheibe N, Sosnowski N, Pinkhasik A, et al. Sonographic evaluation of diaphragmatic dysfunction in COPD patients. *International journal of chronic obstructive pulmonary disease* 2015;10:1925.
39. Seymour JM, Ward K, Raffique A, et al. Quadriceps and ankle dorsiflexor strength in chronic obstructive pulmonary disease. *Muscle & nerve* 2012;46(4):548-54.
40. Shrikrishna D, Patel M, Tanner RJ, et al. Quadriceps wasting and physical inactivity in patients with COPD. *European Respiratory Journal* 2012;40(5):1115-22.
41. Smargiassi A, Inchingolo R, Tagliaboschi L, et al. Ultrasonographic assessment of the diaphragm in chronic obstructive pulmonary disease patients: relationships with pulmonary function and the influence of body composition-a pilot study. *Respiration* 2014;87(5):364-71.
42. Souza RM, Cardim AB, Maia TO, et al. Inspiratory muscle strength, diaphragmatic mobility, and body composition in chronic obstructive pulmonary disease. *Physiotherapy Research International* 2019;24(2):e1766.
43. Sun Q, Liu L, Pan C, et al. Effects of neurally adjusted ventilatory assist on air distribution and dead space in patients with acute exacerbation of chronic obstructive pulmonary disease. *Critical Care* 2017;21(1):126.
44. Vrieze A, De Greef M, Wýkstra P, et al. Low bone mineral density in COPD patients related to worse lung function, low weight and decreased fat-free mass. *Osteoporosis International* 2007;18(9):1197-202.
45. Wallbridge P, Parry SM, Das S, et al. Parasternal intercostal muscle ultrasound in chronic obstructive pulmonary disease correlates with spirometric severity. *Scientific reports* 2018;8(1):1-9.
46. Dos Santos Yamaguti WP, Paulin E, Shibao S, et al. Air trapping: The major factor limiting diaphragm mobility in chronic obstructive pulmonary disease patients. *Respirology* 2008;13(1):138-44.
47. Ye X, Wang M, Xiao H. Echo intensity of the rectus femoris in stable COPD patients. *International journal of chronic obstructive pulmonary disease* 2017;12:3007.
48. Zhang X, Yuan J, Zhan Y, et al. Evaluation of diaphragm ultrasound in predicting extubation outcome in mechanically ventilated patients with COPD. *Irish Journal of Medical Science (1971-)* 2019:1-8.
49. Bhatt SP, Luqman-Arafath T, Gupta AK, et al. Volitional pursed lips breathing in patients with stable chronic obstructive pulmonary disease improves exercise capacity. *Chronic respiratory disease* 2013;10(1):5-10.
50. Andrews SM, Deoghare HV, Mills PK, et al. Pulmonary Rehabilitation Maintenance Program May Prevent Accelerated FEV1 Decline in Patients With COPD. *Clinical Pulmonary Medicine* 2017;24(4):143-48.

51. Alcazar J, Losa-Reyna J, Rodriguez-Lopez C, et al. Effects of concurrent exercise training on muscle dysfunction and systemic oxidative stress in older people with COPD. *Scandinavian journal of medicine & science in sports* 2019;29(10):1591-603.
52. Rocha T, Souza H, Brandao DC, et al. The manual diaphragm release technique improves diaphragmatic mobility, inspiratory capacity and exercise capacity in people with chronic obstructive pulmonary disease: a randomised trial. *Journal of physiotherapy* 2015;61(4):182-89.
53. Nair A, Alaparthi GK, Krishnan S, et al. Comparison of Diaphragmatic Stretch Technique and Manual Diaphragm Release Technique on Diaphragmatic Excursion in Chronic Obstructive Pulmonary Disease: A Randomized Crossover Trial. *Pulmonary Medicine* 2019;2019(6364376)
54. Yamaguti WP, Claudino RC, Neto AP, et al. Diaphragmatic breathing training program improves abdominal motion during natural breathing in patients with chronic obstructive pulmonary disease: a randomized controlled trial. *Archives of physical medicine and rehabilitation* 2012;93(4):571-77.
