# Supplementary material for: Diagnostic and clinical values of non-cardiac ultrasound in COPD: A systematic review
Source: BMJ Open Respir Res. 2020 Sep 25;7(1):e000717. doi: 10.1136/bmjresp-2020-000717 (PMC7520906; doi:10.1136/bmjresp-2020-000717)
Supplement: Supplementary data [file bmjresp-2020-000717supp002.pdf]

Diagnostic and clinical values of non-cardiac ultrasound in COPD: a systematic review

Jaber S Alqahtani[1,2], Tope Oyelade[3], Jithin Sreedharan[1], Abdulelah M Aldhahir[4,2], Saeed M Alghamdi[5,6], Ahmed M Alrajeh[7], Abdullah S Alqahtani[1], Abdullah Alsulayyim[4,6], Yousef Aldabayan[7], Nowaf Y Alobaidi[8,9], Mohammed D. AlAhmari[1]

1 Department of Respiratory Care, Prince Sultan Military College of Health Sciences, Dammam, Saudi Arabia

2 UCL Respiratory, University College London, London, UK

3 UCL Institute for Liver and Digestive Health, London, UK

4 Respiratory Care Department, Faculty of Applied Medical Sciences, Jazan University, Jazan, Saudi Arabia

5 Faculty of Applied Medical Sciences, Umm Al-Qura University, Makkah, Saudi Arabia

6 National Heart and Lung Institute, Imperial College London, London, UK

7 Department of Respiratory Care, King Faisal University, Al Ahsa, Saudi Arabia

8 Institute of Inflammation and Ageing, University of Birmingham, Birmingham, UK

9 Respiratory Therapy Department, King Saud bin Abdulaziz University for Health Sciences, Alahsa, Saudi Arabia

Source of Funding: None.

Word count: 3010

Running Title: Ultrasound Measurements in COPD

Keywords: Ultrasound, COPD, Diagnosis, Prognosis, Diaphragm, Quadriceps

Corresponding author

Jaber S. Alqahtani, MSc ACP CC, ACCP, BSRT, AFHEA, FNIV

UCL Respiratory, University College London, London, Rowland Hill Street, London NW3

2PF, email: Alqahtani-Jaber@hotmail.com

S2. Table 1. Characteristics of included studies on ultrasound measurement of diaphragmatic features in COPD

Study Name	Country/<br>Type of study	Sample size (male= Female=)	Age mean ±SD or range	Targeted population	GOLD severity	Type of assessment	Outcome measures	Result specifically for ultrasound (with numbers and p values)
Yamaguti et al 2008 [1]	Brazil/O	M= 58<br>F= 14	52-70	Stable	Gold 2	Assess intervention effect	To assess diaphragm mobility	COPD patients had less diaphragm mobility (36.5 ±10.9 mm) than healthy individuals (46.3 ±9.5 mm) (P = 0.001) and this s mainly due to air trapping and is not influenced by respiratory muscle strength or hyperinflation
Baria et al 2014 [2]	US/O	M=23<br>F =27<br>(Control Group<br>M=73<br>F=77)	69 ±9.9<br><br>(Control group 50.64 ±17.8)	Stable	Gold 2	Diagnose	To find the standard values for diaphragm thickness and thickening ratio	There was no significant difference in diaphragm thickness or thickening ratio between sides within groups (control subjects or patients with COPD) or between groups, with the exception of the subgroup with severe air trapping (residual volume. 200%), in which the only difference was that the thickening ratio was higher on the left (P value, 004).
Nair et al.2019 [3]	India/RCT	M = 12<br>F = 8	66.85 ± 8.37	Stable	Gold 2	Assess intervention effect	To compare the effects of diaphragmatic stretch and manual diaphragm release technique on diaphragmatic excursion in patients with COPD.	Despite significant differences before and after applying each method, there was no statistically significant difference in diaphragmatic excursion in the comparison of the post intervention values of both techniques.
Okura et al. 2017 [4]	Japan/O	M = 32<br>F = 0	73 ± 7	Stable	Gold 2<br>Gold 3	Diagnose	To assess the contractile capacity of the diaphragm and its relationship with nocturnal oxygen saturation.	NSpO2 mean was positively correlated with (The change in the thickness of the diaphragm) %ΔTdi ($P < 0.001$).
Gorman et al. 2005 [5]	Australia/O	M= 7<br>F = 5	63 ± 8	Stable	Gold 4	Diagnose	To estimate the diaphragm length after lung volume reduction surgery	Increased diaphragm length resulted in lower motor unit firing rates and reduced breathing effort, and this is likely to contribute to improved quality of life and exercise performance after LVRS.
Gorman et al. et al 2002 [6]	Australia/O	M = 10<br>F=0<br><br>Control (10)	68 ± 9	Not stable, (Had recurrent hospital admissions)	Gold 4	Diagnose	To estimate the diaphragm length during tidal breathing in COPD	Although the diaphragm length during tidal breathing is shorter at FRC in patients with COPD, its motion and change in length during tidal breathing is like that in control subjects.
Grosu et al. 2017 [7]	US/O	COPD=8<br>M = 33<br>F =24	65.7 ± 16.7	Not Stable (mechanically ventilated)	No data	Diagnose	To estimate the changes in diaphragm thickness in intubated patients.	➢ Subjects without COPD had thinner diaphragm at baseline than those with COPD (0.22 ±0.07 vs 0.26 ± 0.06, p value, 0.03).<br>➢ No significant association between the rate of thinning and diagnosis of COPD, (P value 0.36).
El Aziz, Amal et al. 2017 [8]	Egypt/O	50<br>COPD<br>M=30<br>F=10<br><br>Control 10	54.3 ± 6.49<br><br>(40=-70 Years)	Stable	All 4 categorie s	Diagnose	To assess the diaphragmatic thickness and excursions in COPD patients.	➢ Diaphragmatic thickness at different lung volumes (TDRV, TDFRC and TDTLC), and excursion all are decreased in COPD than control. Highly significant positive correlation was found between diaphragmatic thickness at different lung volumes (TDRV, TDFRC and TDTLC) diaphragmatic thickenings and excursion with the spirometric finding as FEV1, MIP and MEP

Yamaguti WP et al. 2012 [9]	Brazil/RCT	M=22<br>F= 8	50-80 years	Stable	Gold 2<br>Gold 3	Assess intervention effect	Improvements in abdominal motion during natural breathing and in diaphragmatic mobility, from baseline to post-DBTP, were used	➢ DBTP in patients with COPD leads to improvements in abdominal motion during NB and in functional capacity. We also showed that patients with a baseline level predominance of costal breathing and worse diaphragmatic mobility experienced a greater improvement in abdominal motion
Corbellini et al. 2018 [10]	Italy/O	COPD<br>M=35<br>F=11<br><br>Control (16)	72 ± 10	Stable	Gold 2<br>Gold 3<br>Gold 4	Assess intervention effect	To correlate the diaphragmatic mobility loss to COPD severity. And estimate the diaphragmatic mobility after pulmonary rehabilitation.	➢ The diaphragmatic mobility demonstrated significant differences between the subjects with COPD and the healthy controls. During resting breathing, mobility was statistically and clinically higher, especially at the beginning of the rehabilitation program, without improvements thereafter.<br>➢ Diaphragmatic mobility during deep inspiration was lower than in the healthy controls, but improved after rehabilitation, and those improvements were followed by improvements of the inspiratory capacity. Second, diaphragmatic mobility reduction was correlated to loss in lung function.
He L et al. 2014 [11]	China/O	124<br><br>COPD<br>M=52<br>F= 6	66.54 ± 9.43	Stable	Gold 1<br>Gold 2<br>Gold 3<br>Gold 4	Diagnose	To compare diaphragmatic motion measured by M-mode ultrasonography of patients with CPFE, IPF and COPD	➢ COPD patients, especially those with severe COPD, showed significantly lower diaphragmatic motion than IPF patients or healthy controls. Right diaphragmatic motion during deep breathing was negatively correlated with emphysema scores (r=-0.60, p value, 0.001.<br>➢ M-mode ultrasonographic evaluation of diaphragmatic motion during deep breathing may be a useful tool in diagnosing CPFE and in discriminating CPFE patients from IPF or COPD patient.
Eryüksel et al. 2017 [12]	Turkey/O	M=47<br>F=6	62.6 ± 10.1	Stable	Gold 1<br>Gold 2<br>Gold 3<br>Gold 4	Diagnose	To examine the value of this measurement in identifying patients with COPD who are at high risk for the development of symptoms and exacerbations	➢ Diaphragmatic thickness fraction measurements based on diaphragmatic ultrasound assessment in subjects with COPD seemed to be unable to identify subjects at high risk of symptoms and exacerbations.<br>➢ Frequency of exacerbations (P=0.46) ABCD symptom evaluation (P=0.51)
Ogan et al. 2019 [13]	Turkey/O	COPD 34<br>M=29<br>F=5<br><br>Control (34)	71.0 ± 9.2	Stable	Gold 3	Diagnose	To determine the muscle thickness of the diaphragm and the usefulness of clinical practice in patients with COPD.	➢ There was no significant difference between the patients and control groups in diaphragmatic thickness.<br>➢ Thickness (R) in COPD vs control:<br>2.74 ± 0.4 vs 2.98 ± 0.8 (p=0.134)<br>Thickness (L) in COPD<br>2.77 ± 0.4 vs 2.70 ± 0.7, (p=0.647).<br>➢ There was also no significant difference between diaphragmatic thickness and COPD severity, respiratory function (P = 0.410), and frequency of exacerbations (P = 0.881) and mMRC (P = 0.667).
Bhatt et al. 2009 [14]	India/O	M=10<br>F = 3	52.2± 6.8	Stable	Gold 3	Assess intervention effect	To examine changes in respiratory dynamics by using ultrasonography in patients with COPD sitting, leaning forward with hands supported on the knees (tripod position)	➢ There was no statistical significance between the three postures in the degree of excursion of the diaphragm<br>➢ Tidal Excursion in Sitting: 16.1±5.9, Supine 20.1±6.8, Tripod 16.6±6.2 (P=0.22).<br>➢ Forced Excursion in Sitting: 33.9±11.0, Supine 43.1±19.6, Tripod 37.4±17.1 (P=0.35).

Evrin et al. 2019 [15]	Turkey/O	M=83<br>F=18<br><br>COPD 61<br>40 control	58-72	Stable	Gold 1<br>Gold 2<br>Gold 3<br>Gold 4	Diagnose	To determine the use of point-of care ultrasound in evaluating the disease status and outcomes in COPD patients.	➢ Five point-of-care US measurements, Lung Sil R, Lung Sil L,Ant B-Mode R, Ant M-Mode R, and Ant M-Mode L, were significantly different among the patient groups (P<0.001 for each)<br>➢ FEV_1 positively correlated and annual number of exacerbations were negatively correlated with US findings.
Zhang et al. 2019 [16]	China/O	M= 27<br>F= 10	74.5 ± 7.5	Stable<br>(On MV, Ready for extubation)	----	Assess intervention effect	To estimate the diaphragmatic activity and its variation in predicting extubation outcome in mechanically ventilated COPD patients.	➢ (AUC-ROC) of DE30 and ΔDE30−5 (the variation between 30 and 5 min) were 0.762 and 0.835; a cut-off value of DE30>1.72 cm and ΔDE30−5> 0.16 cm were associated with a successful extubation with a sensitivity of 76% and 84%, a specificity of 75% and 83.3%, respectively.<br>➢ Combination of diaphragmatic excursion (DE30 and ΔDE30−5) estimated by the bedside ultrasound could improve the predictive value and could be used as the predictor of extubation outcome in mechanically ventilated patients with COPD.
Sun et al. 2017 [17]	China/O	M = 13<br>F = 2	78.6 ± 7.01	Stable<br>(Mechanically ventilated COPD)	-----	Assess intervention effect	Ultrasonic assessment of the diaphragm activities during NAVA and PSV mode during the weaning phase of mechanical ventilation.	➢ The diaphragm activity in patients with AECOPD decreased when the support level increased for both PSV and NAVA. However, the diaphragm activities were higher during NAVA compared to the same sup-port level during PSV.<br>➢ There was a positive correlation between the diaphragm activity and ventilation distribution in the most dependent regions (ROI4, R^2= 0.56, P< 0.01).
Priori et al. 2013 [18]	Italy/O	23 COPD<br><br>12 Control	66.4 ± 6.9	Stable	Gold 3	Assess intervention effect	To assess the kinematics of the diaphragm by ultrasonography	➢ In COPD patients, the Diaphragm displacement estimated by ultrasonography was 23.6 ± 9.3 mm while seated and 17.5 ± 5.7 mm supine (P = 0.05). COPD patients showed significantly greater diaphragmatic displacement seated compared with control subjects (P = 0.01), but when supine the degree of displacement was similar in both groups.<br>➢ Rib cage paradox was noticed in approximately one-half of the COPD patients while seated but was not related to impaired diaphragm motion. In the supine posture, the rib cage paradox disappeared, suggesting that, in this posture, diaphragm mechanics improves.
Rocha et.al. 2015 [19]	Italy/RCT	M=14<br>F=5	71 years	Stable	Gold 3	Assess intervention effect	To evaluate diaphragmatic mobility	➢ The Manual Diaphragm Release Technique produced statistically significant improvements in diaphragmatic mobility, 6-minute walking distance and inspiratory capacity in people with COPD.<br>➢ The improvement in diaphragmatic mobility showed moderate correlation with abdominal volume during inspiratory capacity manoeuvres.
Elsawy et al. 2017 [20]	Egypt/O	COPD<br>M= 100<br>F = 0<br><br>Control<br>100	63.61 ± 5.6	Stable	Gold 1<br>Gold 2<br>Gold 3<br>Gold 4	Diagnostic	To assess the diaphragm thickness and its impact on disease severity in COPD patients.	➢ The right (25.3 ± 5.1. vs 30.8 ±5.4) and left thickness traction (31.3 ±7.2 vs 34.1 ±8.5) were significantly lower in COPD group than control group.<br>➢ The right and left thickness fraction were significantly decreased through progression of COPD (mild, moderate, severe and very-severe grades) (p = 0.020, 0.002 respectively).<br>➢ The right and left thickness fraction were positively correlated with BMI, FEV1/FVC, FEV_1% and PaO2, while it was negatively correlated with PaCO2, which indicates that diaphragm

								dysfunction in COPD patients is the main contributor to neuromuscular respiratory failure.
Paulin et al. 2007 [21]	Brazil/O	COPD= 54<br>M=42<br>F=12<br>Control=20	62.15± 8.06	Stable	Gold 3	Diagnostic	To evaluate the influence diaphragmatic mobility has on exercise tolerance and dyspnea in patients with COPD.<br>.	➢ The COPD patients presented less diaphragmatic mobility than did the controls (36.27± 10.96 mm vs. 46.33±9.46 mm)<br>➢ Diaphragmatic mobility was found to correlate with RV(r=-0.60;p<0.000), IC (r=0.41;p<0.002), the RV/TLC ratio (r=-0.72;p<0.000), and the IC/TLC ratio(r=0.55;p<0.000<br>➢ Diaphragmatic mobility also correlated significantly with performance on the 6MWT (r=0.38; p<0.005) and dyspnea score (r=-0.36; p<0.007).
Kang et al. 2011 [22]	South Korea/O	M=31<br>F=6	69.1 ± 9.0	Stable	Gold 2,3,4	Diagnostic	The purpose of the study was to investigate the relationship between diaphragm mobility and pulmonary function parameters, as well as that between arterial blood gas values and diaphragm mobility in COPD patients.	➢ Diaphragm mobility (mm)19.8 ± 7.5<br>➢ Diaphragm mobility correlated with the (FEV_1: r = 0.415, P = 0.011) and pulmonary hyperinflation (RV: r = -0.501, P = 0.021; TLC: r = -0.281, P = 0.030; RV/TLC: r = -0.527, P = 0.001).<br>➢ It also correlated significantly with (FVC: r = 0.302, P = 0.029; MVV: r = 0.481, P = 0.003).<br>➢ Negative correlations between diaphragmatic mobility and $PaCO_2$ (r = 0.373, P = 0.030).
Souza et.al. 2019 [23]	Brazil/O	M=13<br>F=8	65.80 ± 7.00	Stable	Gold 2,3,4	Diagnostic	To evaluate inspiratory muscle strength, diaphragmatic mobility and body composition in COPD subjects and to correlate these variables	➢ Subjects with inspiratory muscle weakness had reduced diaphragmatic mobility compared to those without, (76.64 ± 12.87 vs 62.53 ± 7.92; P=0.007)<br>➢ Maximal inspiratory pressure exhibited a weak positive relationship to diaphragmatic mobility (r= 0.496, p= 0.022)<br>➢ Inspiratory muscle weakness had repercussions on the mobility of the diaphragm muscle
Lim et al. 2019 [24]	South Korea/O	M=10<br>F= 0	79.8 ± 8.1	acute exacerbation	Gold 3,4	Diagnostic	To investigate the changes in diaphragmatic function during acute exacerbation of COPD by US	➢ The significant increase in the diaphragmatic thickness from the initial (at exacerbation) to the follow-up (stable phase) values (80.1 ± 104.9 mm vs. 159.5 ± 224.6 mm, p = 0.011);<br>➢ Diaphragmatic excursion did not vary significantly between the initial and follow-up values (22 ± 6 mm vs 23 ±12 mm).<br>➢ The change in excursion between the stable and exacerbation periods was positively correlated with time to the next exacerbation (r=0.89; p=0.04) and negatively correlated with the time taken to recover from the exacerbation (r=-0.77; p=0.07).<br>➢ Diaphragmatic dysfunction occurs during AECOPD, and patients showing greater improvement of function during recovery will likely have delayed appearance of future exacerbations.
Abbas et.al 2018 [25]	Egypt/O	M = 34<br>F =16	61.9±7.47	Stable<br><br>Mechanically Ventilated	Gold 2, 3	Diagnostic	To validate the D-RSBI as a new predictor of weaning outcome in patients with AECOPD and to compare its accuracy with that of the traditional RSBI.	➢ D-RSBI (RR/DD) is superior to the traditional RSBI (RR/VT) in predicting weaning outcome in AECOPD patients<br>➢ The areas under the ROC curves for D-RSBI and RSBI were 0.97 (p<0.001) and 0.67 (p<0.06), respectively.<br>➢ DD (mm), 16.57±2.4 vs 9.23±2.42, (P= <0.001)<br>➢ D-RSBI = Successful weaning, 1.43±0.32 vs failed weaning 3.27±0.84 (P= <0.001)
McKenzie et al. 2000 [26]	Australia/O	M = 9<br>F =0	65 (10)	Stable	Gold 4	Diagnostic	To measure the Diaphragm length (LDi) via measurements of transverse diameter of the rib	➢ Diaphragm dimensions in patients with severe COPD can be predicted from measurements of LZapp and DRc.<br>➢ The findings validate the use of non-invasive

							cage (DRc) and the length of the zone of apposition (LZapp) in COPD patients	measures of LZapp (e.g. using ultrasonography) and DRc (e.g. using magnetometers) to estimate LDi in COPD patients.
Jain et al. 2019 [27]	India/O	COPD 48 M=27 F=21 Control 22	Not reported	Stable	Gold 1,2,3,4	Diagnostic	to compare the changes in the diaphragm in COPD patients in Indian population with the help of ultrasound-guided examination.	➢ Mean diaphragmatic thickness during inspiration and expiration and zone of opposition were significantly decreased in patients with small airways, mild to moderate obstruction and increased in cases with severe airway obstruction as compared to controls.<br>➢ Movement of diaphragm was significantly more in cases with moderate to severe obstruction as compared to controls
Smargiassi et al. 2014 [28]	Italy/O	M=23 F=9	71.8 ± 8.2	Stable	Gold 1,2,3	Diagnostic	To estimate the diaphragm thickness to evaluate the relationships between echographic measurements, parameters of respiratory function and body composition data.	➢ TD at different lung volumes was closely related to IC, vital capacity (VC) and TLC, showing TDTLC to have the closest relationships with IC and VC (r= 0.42 and p = 0.0001 in both cases).<br>➢ TDTLC was related to all indices of air trapping: directly to IC/TLC (p = 0.01), inversely to FRC/TLC and to RV/TLC (p = 0.01 and p = 0.02, respectively<br>➢ FFM showed significant direct correlations with all TD measurements (i.e. TDFRC, TDTLC and TDRV), with closer relationships with TDFRC and TDTLC (r= 0.39 and p = 0.0002 and r= 0.32 and p = 0.0008, respectively).<br>➢ Echographic measurements of TDTLC and thickening at TLC might be a useful tool to estimate lung hyperinflation, especially when adjusted for FFM
Cimşit et al. 2016 [29]	Turkey/O	M=47 F = 6	62.57 ± 9.8	Stable	Gold 2,3,4	Diagnostic	Ultrasound evaluation of the diaphragm thickness and to determine the relationship between pulmonary function tests, and symptom scores	➢ Mean thickness value was 2.3 mm in females and 2.06 mm in males<br>➢ There was a moderate correlation between diaphragmatic muscle thickness and %FEV_1 in mild COPD patients (r=0.62, p=0.017<0.05). No significant difference in diaphragmatic thicknesses of GOLD subgroups was found. There were no correlations between diaphragmatic muscle thickness, symptom scores, BMI, age, and gender.
Marchioni et.al 2018 [30]	Italy/O	M=38 F=37	78 (71–86)	Exacerbation	Gold 1,2,3,4<br><br>(Categorised based on the previous history)	Diagnostic	Correlations between the change in diaphragm thickness (ΔTdi) < 20%, NIV failure and other clinical outcomes were investigated.	➢ Early and noninvasive US assessment of DD during severe AECOPD is reliable and accurate in identifying patients at major risk for NIV failure and worse prognosis<br>➢ Diaphragmatic thickness ΔTdi < 20% showed better accuracy in predicting NIV failure than baseline pH value and early change in both arterial blood pH and partial pressure of carbon dioxide following NIV start (AUCs 0.84 to DTdi < 20%, 0.51 to pH value at baseline, 0.56 to early change in arterial blood pH following NIV start, and 0.54 to early change in partical pressure of carbon dioxide following NIV start, respectively; p < 0.0001).
Crimi et.al. 2018 [31]	Italy/O	M=23 F=2	70.6±6.5	Stable	Gold 1,2,3,4	Assess intervention effect	To estimate the role of US assessment of diaphragm function and change of rectus femoris area in COPD patients undergoing a PR program and in the detection of post rehabilitation outcomes.	➢ A correlation was found between the intraindividual percentage of change in the diaphragm length of zone of apposition at functional residual capacity (ΔLzapp%) and the change in 6-minute walking distance (6MWD) after PR (r=0.49, P=0.02)<br>➢ ΔLzapp% was significantly higher in patients with improved 6MWD and COPD Assessment Test (CAT) score (mean rank=12.03±2.57 vs 6.88±4.37; P=0.02).

								➢ Diaphragm US assessment represents a useful prognostic marker of PR outcomes in COPD patients.<br>➢ Diaphragmatic excursion (mm)–<br>Quiet breathing<br>Pre PR - 23 (16–27)<br>Post PR 23 -27 (22–31), P= 0.001<br>Deep breathing<br>Pre PR/ 36 (25–53)<br>Post PR/ 50 (35–58), P= 0.001<br>➢ Rectus femoris area (m^2): pre 4 (3–4) vs post 4 (3–5), p <0.001
Bhatt et.al. 2013 [32]	USA/RCT	M=10<br>F=4	53.1 ± 7.4	Stable	Gold 2,3,4	Assess intervention effect	To measure the diaphragmatic movement during tidal and FVC Maneuver to estimate the changes in exercise capacity	➢ There was a significant correlation between improvement in 6MWT distance and increase in diaphragmatic excursion during forced breathing when measured by US (r=0.59, p=0.03).<br>➢ Tidal excursion (cm), Pre-PLB 2.01+0.7 vs Post 2.23+0.5<br>➢ PLB increases functional exercise tolerance. This effect could be mediated by a reduction in RR and increased diaphragmatic movement.
Andrews et al. 2017 [33]	Canada/RCT	M=67<br>F= 33	76.3 ± 11.9	Stable	GOLD 1, 2, 3	Assess intervention	To assess the effect of EMT on lung function and outcomes in patients that completed PR.	➢ Ultrasound assessment revealed no difference in the RFcsa or diaphragm thickness between the EMT and control group<br>➢ US diaphragm in EMT and control (cm ± SD 0.3 ± 0.1 vs 0.3 ± 0.) respectively.<br>➢ US rectus femoris in EMT and control (cm ± SD 2.8 ± 0.5 vs 2.9 ±0.7) respectively.
Scheibe et al. 2015 [34]	Germany/O	COPD 60<br>Control 20		Stable	Gold 2,3,4	Diagnostic	To compare sonographic measurement of the lung silhouette movement and investigate the correlation between both measures and lung function parameters in COPD patients	➢ Ultrasound measurement of the lung silhouette correlated strongly with direct measurement of the right hemidiaphragmatic movement (r=0.85).<br>➢ The up and downward movement of the lung silhouette measurement also correlated strongly with FEV_1 (r=0.83, P<0.05).<br>➢ The sonographic measurement of the movement of the lung silhouette is an easy way to establish diaphragmatic dysfunction in COPD patients; it can be done in all patients with reliable results for the right and the left hemidiaphragm
Maynard-Paquette et al. 2020 [35]	Canada/O	M=20<br>F= 0	66±6	Stable	Gold 1,2,3,4	Diagnostic	To evaluate the relationship between quadriceps size, and symptoms, lung function and diaphragm contractility in COPD patients using ultrasound.	➢ Mean Qcsa, Qthick and Qci were 336±145 mm2, 1.55±0.53 cm and 64±16%, respectively, and mean TFdi was 91±36%<br>➢ Qci was significantly correlated with FFMI (rho=0.59, p=0.001), TFdi (rho=0.41, p=0.008), FEV1 (rho=0.43, p=0.001) but not with age (rho=0.18, p=0.28).<br>➢ Qci was significantly correlated to CAT score (rho=−0.47, p=0.002), even when controlled for FEV1, and was lower in patients with an mMRC score ≥2 (55±15 vs 70±14%, p=0.002).<br>➢ Q_{CSA} and Q_{ci} were significantly lower in patients with regular exacerbations<br>➢ Only FFMI and TFdi were found to be significantly related to lower Qci values.<br>➢ US evaluation of the quadriceps contractile index is feasible and related to disease severity, clinical symptoms, and exacerbation history and diaphragm contractility.

Abbreviations: %ΔTdi: percentage changes in diaphragm thickness; 6MWD: six minute walk distance; AECOPD: acute exacerbation of chronic obstructive pulmonary disease ; AUC-ROC: Area under the curve, receiver-operating characteristic; BMI: body mass index ; CAT: COPD Assessment Test; COPD: chronic obstructive pulmonary disease; CPFE: Combined pulmonary fibrosis and emphysema; DBTP: diaphragmatic breathing training program; DD: diaphragmatic dysfunction; DE: diaphragmatic excursion; DRc: diameter of the rib cage ; D-RSBI: diaphragmatic rapid shallow breathing index ; EMT: exercise maintenance therapy; F: female ; FEV_1: Forced expiratory volume in one second; FFM: fat-free mass ; FRC: functional residual capacity; FVC: forced vital capacity; GOLD: global initiative for chronic obstructive lung disease; IC: inspiratory capacity ; IPF: interstitial pulmonary fibrosis; LDi: diaphragm length ; LVRS: lung volume reduction surgery; LZapp : length of the zone of apposition; M: male ;MEP: maximum expiratory pressure; MIP maximum inspiratory pressure; mm: Millimetre; mMRC: modified medical research council; NAVA: neurally adjusted ventilatory Assist; NB: normal breathing; NIV: noninvasive ventilation; NSpO2: nocturnal oxygen saturation ; O: observational ; PaCO2: carbon dioxide pressure in arterial blood; PaO2: oxygen pressure in arterial blood; PR: pulmonary rehabilitation; PSV: pressure support ventilation; Qci: quadriceps contractile index ; Qcsa: quadriceps cross-sectional area; Qthick: quadriceps thickness ; RCT: randomised control trial ; RFcsa: rectus femoris cross-sectional area ; RR/DD: respiratory rate/ diaphragmatic displacement; TD: diaphragm thickness; TDFRC: diaphragm thinness at functional residual capacity ; TDRV: diaphragm thinness at residual volume ; TDTLC: diaphragm thinness at total lung capacity; TFdi: thickening fraction; US: ultrasound ; VC: vital capacity

S2. Table 2 – Characteristics of included studies on ultrasound measurement of muscles features and bone mineral density in COPD

Study Name	Country /Type of study	Sample size (male/female)	Age mean ±SD or range	Targeted population	COPD GOLD	Location of use	Type of assessment	Outcome measures	Result specifically for ultrasound (with numbers and p values)
Maddocks et al. 2014 [36]	UK/O	COPD M=10 F=7<br>Healthy M= 16 F = 22	68 (10)	Stable	GOLD 3	Tibialis anterior cross-sectional area	Diagnose	To assess change in strength, loss and change in the composition of skeletal muscles in patients with COPD.	➢ Reduction in ankle dorsiflexor muscle strength (ADMVC, 100 HzAD) in COPD patients compared to age-matched and younger healthy subjects when normalized to TA_{CSA} (P<0.01). TA_{EI} increased in COPD patients compared healthy young (P=0.0008) and elderly (P=0.025) subjects.<br>➢ There were no significant differences in TACSA between healthy young, healthy elderly and COPD patient groups<br>➢ In all participants ADMVC and 100 HzAD positively correlated with TA_{CSA} (r=0.78, P<0.0001) and negatively correlated with TA_{EI} (r=−0.46, P<0.0005).<br>➢ Muscle Infiltration of non-contractile tissue is related to loss in muscle strength observed in COPD patients.
Greening et al 2015 [37]	UK/O	M=78 F=113	71.6 (9.1)	Exacerbated	Gold 1,2,3,4	Quadriceps RFcsa	Diagnose	To determine whether muscle (quadriceps) function as assessed by ultrasound can predict hospital readmission at 1 year or death in COPD patients	➢ Quadriceps RFcsa was associated with readmission or death (OR=0.34, 95% CI= 0.17–0.65, P= 0.001), and hospitalization in the previous year (OR, 4.82; 95% CI, 2.42–9.58; P,0.001).<br>➢ Subjects in the smallest muscle group were more likely to be readmitted or die (as expected) but also had more days in hospital over the subsequent 12 months.<br>➢ Qcsa, cm2:4.76 (1.41)
Ye X et al. 2017 [38]	China/O	COPD M=27 F=23<br>Control 21	65.76±8.07	Stable	Gold 1,2,3,4	Rectus femoris	Diagnose	To investigate the relationship between rectus femoris echo intensity and COPD (HRQoL) using ultrasound.	➢ EI was significantly higher in GOLD I/II/III COPD than in non-COPD subjects (P=0.019/ P=0.005/ P<0.0001).<br>➢ QMT (mm) was lower in GOLD III-IV COPD compared to non-COPD and GOLD I COPD subjects (P=0.000 and P=0.001).<br>➢ RF_{CSA} (cm^2) was lower in GOLD III-IV COPD (3.99±1.16) compared to non-COPD (7.07±1.78) and GOLD I COPD (6.84±2.67), (P=0.006 and P=0.01).<br>➢ In stable COPD patients QMT, RFcsa, FACITF and 6MWD showed positive correlation with lung function, while EI showed significant negative correlation with lung function (r=−0.413, P<0.01).
Alcazar et al. 2019 [39]	Spain/RCT	M=24 F=5	77.7 ± 7.9	Stable	Gold 1,2,3,4	Vastus lateralis (VL) muscle	Assess intervention effect	To assess the effects of ET on systemic oxidative stress and limb muscle dysfunction in older people with COPD via measurement of plasma protein carbonylation and muscle ultrasonography respectively.	➢ In ET subjects there was a significant change in mid-thigh muscle CSA (+4%), VL muscle thickness (+11%) and pennation angle (+19%).<br>➢ Also, protein carbonylation decreased in the ET group compared to the control group (-27%, P<0.05) and correlated with changes in Muscle size and pennation angle (r = −0.44 to −0.57), exercise capacity (= −0.46). muscle strength (r = −0.45) and sit-to-stand performance (r = 0.60).
Vrieze et.al. 2007 [40]	Netherlands/O	M=62 F=53	60.0±10.8	Stable	Gold 2,3,4	Bone mineral density	Diagnostic	To investigate the prevalence and predictors of abnormal	➢ The prevalence of abnormal BMD in COPD rises from 27% in GOLD II to 75% in GOLD IV COPD.

								BMD in COPD by ultrasound densitometer.	➢ Risk factors of low BMD abnormal (T-score < −1.0) are FFM, BMI and FEV_1.<br>➢ GOLD IV COPD patients have 7.6 times greater risk of abnormal BMD than GOLD II patients (95% CI = 2.4 – 24.3, P<0.05).<br>➢ Advanced COPD, low BMI and low FFM are risk factors for the presence of low BMD.
Wallbridge et.al 2018 [41]	Australia /O	M=16<br>F=4	62.3–78.8	Stable	Gold 2,3,4	Parasternal intercostal muscle	Diagnostic	To investigate the validity of parasternal intercostal muscle ultrasound as an alternative to computed tomography-based measurement and correlation of the ultrasound measurements with COPD severity	➢ In stable COPD, ultrasound measurements of parasternal intercostal muscle thickness and density (quality) is a reliable technique for clinical use.<br>➢ In general, ultrasound-measured intercostal thickness correlated with FEV_1 (r=0.33), quadriceps thickness (r=-0.32).<br>➢ Ultrasound-measured mean echogenicity (indicating poorer muscle quality) showed a moderate negative correlation with FEV1% predicted, (r = −0.32)
Seymour et.al 2012 [42]	UK/RCT	COPD<br>M=12<br>F=8<br>Control 18	72 ± 8	Stable	Gold 3, 4	RF_{CSA}, and (TA_{CSA})	Diagnostic	To assess difference in strength and size of skeletal muscles in CODP and elderly group	➢ Reduced quadricep size and strength in COPD patients (mean MVC difference: -10.9 kg (95%CI= -17.1kg to -4.8kg, P<0.01 and mean RF_{CSA} difference: -119 mm^2, 95% CI=-180 mm^2 to -58 mm^2, P<0.01).<br>➢ No significant difference in FFM index, TA_{CSA} or ankle dorsiflexor strength recorded.
Coratella et.al. 2018 [43]	Italy/O	M= 35<br>F = 0	65 ± 4	Stable	Gold 3, 4	Vastus lateralis (VL) muscle	Diagnostic	To compare muscle strength and architecture in COPD versus healthy subjects using ultrasound.	➢ The eccentric peak-torque of COPD and control group at low (2.57 ± 0.55 and 2.80 ± 0.60 $N·m·kg^{-1}$, p=0.128 respectively) and high (2.44 ± 0.51 and 2.58 ± 0.46 $N·m·kg^{-1}$, p=0.259 respectively) angular velocity was similar.<br>➢ Greater vastus lateralis pennation angle and muscle thickness were found in CON vs COPD, although no difference was observed in fascicle length<br>➢ FEV_1 and FEV_1/FVC correlated negatively (r=-0.465 and r=-0.414 respectively) and moderately (p<0.05) with eccentric-to-concentric peak-torque ratio and peak-torque respectively.
Kaneko et.al 2008 [44]	Japan/O	COPD<br>M = 15<br><br>Control 15	71±5.2	Stable	Gold 3, 4	Abdominal Muscles	Diagnostic	To assess the relationship between resting expiratory activity of the lateral abdominal muscle and exercise tolerance in COPD patients compared to healthy control	➢ Overall, Tdif of the lateral abdominal muscle was higher (20.7±5.4% vs 10.2±4.4%) and distance walked was lower (278±172m vs 571±129m) in COPD patients compared to with the healthy subjects (all P<0.05).<br>➢ Significant negative correlation was observed between Tdif and distance walk (r=–0.58, p<0.05).<br>➢ No significant correlation between Tdif and FEV_1 in both the COPD and control groups.<br>➢ Expiratory muscle activity during breathing is more linked with exercise intolerance than airflow limitation.
Górka et al 2016 [45]	Poland/ O	M = 24<br>F = 9	66.8 ± 8.8	Stable	Gold 2, 3,4	Bronchial wall layer thickness	Diagnostic	To assess the relationship between emphysema severity, bronchial wall thickness using EBUS, and markers of remodelling (MMP-9) in BALF in COPD patients	➢ Matrix metalloproteinase 9 (MMP-9) significantly higher in severe COPD and negatively correlated with FEV_1.<br>➢ Thickness of mucosa/submucosa and smooth muscle positively correlated with BALF TGF-β (r =0.366, P=0.046 and r=0.425, P=0.02), while the mucosa/submucosa thickness negatively correlated with neutrophil elastase (r=–0.508, P=0.004). No correlation between the BALF markers and emphysema score.<br>➢

Alqahtani JS, *et al. BMJ Open Resp Res* 2020; 7:e000717. doi: 10.1136/bmjresp-2020-000717

Cruz-Montecinos et al.2016[46]	Spain/O	M = 11<br>F =9	70 ± 7	Stable	Gold 3,4	Rectus femoris, Vastus intermedius and anterior compartment of the right thigh	Diagnostic	To assess the relationships between 6MWT and MVCQ and quadriceps thickness and echo intensity in COPD patients	➢ Positive correlation between MVCQ and thickness of rectus femoris and vastus intermedius (r=0.427; P=0.030; r= 0.469; P=0.018, respectively). Negative correlation between MVCQ and echo intensity of rectus femoris and vastus intermedius (r=–0.500; P=0.012; r=–0.482; P=0.016).<br>➢ 6MWT and MVCQ not correlated (r=0.319; P=0.085).<br>➢ Accordingly, 70% 0f variance in 6MWT result is explained by vastus intermedius thickness and echo intensity and the echo intensity of rectus femoris.<br>➢ In COPD, exercise capacity and quadriceps force are linked to quantity and quality of quadriceps muscle.
Nijholt et al. 2019 [47]	Netherlands/O	M = 19<br>F = 25	59.8 ± 8.6	Stable	Gold 4	Rectus femoris size	Diagnostic	To correlate ultrasound measured rectus femoris size with fat-free mass and muscle function in patients with COPD	➢ RF_{CSA} and thickness positively correlated with FFMI (r = 0.57, P<0.001; r= 0.53, P=0.003, respectively) and HGS (CSA r = 0.58, p < 0.001, thickness r = 0.48, p = 0.009).<br>➢ No significant correlations between RF-thickness, CSA, and leg muscle power (r= 0.33, p=0.091; r=0.35, p=0.073, respectively).<br>➢ Furthermore, no correlation between RF size and maximal exercise capacity was observed (thickness r = 0.21, p=0.297, CSA r=0.22, p = 0.274).
Navarro-Cruz et.al. 2019 [48]	Spain/O	COPD<br>M= 26<br>F = 0<br>Control 10	79 ± 7	Stable	Gold 3, 4	Vastus lateralis muscle	Assess intervention effect	To assess the effect of SSC on portions of force-velocity relationship and the link with physical function in adults with and without COPD.	➢ Lower physical function and concentric maximal muscle power were lower in COPD patients compared to non-COPD subjects (P<0.05).SSC-induced potentiation at 50-100% and 80-100% of V0 negatively correlated with physical function (r=–0.40 to –0.50) and VL thickness and pennation angle (r=0.43–0.52) (all p<0.05).<br>➢ SSC-induced potentiation is higher in adults with COPD than in non-COPD adults and is associated with lower physical function, VL thickness and pennation angle values.<br>➢ SSC-induced potentiation is a possible interventional candidate in older adults with decreased concentric muscle actions.
Shrikrishna et al. 2012 [49]	UK/O	COPD<br>M=88<br>F= 35<br>Control 31	67 ± 9	Stable	Gold 1, 2, 3, 4	Rectus femoris	Assess intervention effect	To investigate possible quadricep muscle bulk reduction in mild to advanced COPD using ultrasound and to assess its relationship with physical activity.	➢ RFCSA was reduced in COPD GOLD I-IV patients compared with healthy subjects (P<0.001).<br>➢ Physical activity was also reduced in GOLD I-IV COPD patients compared with healthy adults (P<0.05).<br>➢ While in COPD GOLD I RFCSA was linked with physical activity, only lung spirometry was the only predictor of physical activity in COPD GOLD II-IV.<br>➢ Quadriceps muscle loss is present in mild to advanced COPD and independently predicts physical activity in COPD GOLD I.<br>➢ USRFCSA has potential as a physiological biomarker in COPD, and the identification of these patients may guide early lifestyle and therapeutic interventions
Menon et al. 2012 [50]	UK/O	COPD<br>M=27<br>F=18<br><br>Control 19	68.2 ± 8.2	Stable	Gold 1, 2, 3, 4	Lower limb muscle mass	Assess intervention effect	Ultrasound assessment for Quantifying the improvements in lower limb or quadriceps muscle mass following resistance training in COPD patients.	➢ Resistance training resulted in significant increase in thigh lean mass, RF_{CSA} and quadriceps muscle thickness in both COPD (+5.7%, +21.8%, +12.1% respectively) and non-COPD (+5.4%, +19.5%, +10.9% respectively) subjects.<br>➢ Effect size for RF_{CSA} (COPD=0.77; Healthy=0.83) and quadriceps muscle thickness (COPD=0.36; Healthy=0.7) changes were greater than

									for thigh lean mass, (COPD=0.19; Healthy=0.26) changes following resistance training.<br>➢ Ultrasound measurement of the quadricep can be used to detect muscle mass changes in response to resistance training.
Ramírez-Fuentes et al. 2019 [51]	Spain/O	COPD M=18 F = 0 Control 17	67.5 (SD 9.0)	Stable	Gold 3, 4	Rectus femoris muscle	Diagnostic	To determine the relationship of the size of the rectus femoris muscle, assessed by ultrasonography, with parameters of muscle strength and body composition used in diagnosis of sarcopenia in COPD patients undergoing rehabilitation.	➢ Reduced RF_{CSA} in COPD patients (4.3±1.05 cm^2) compared with healthy subjects (5.6±1.25 cm^2).<br>➢ Also reduced quadriceps strength and FFM was observed.<br>➢ RF_{CSA} also correlated with quadriceps strength (r=0.497, P=0.036) and FFM (r=0.584, P=0.011).<br>➢ After adjusting for most other factors, COPD patients reported a lower muscle strength (-7.44kg, P=0.014) compared with healthy subjects.<br>➢ RF_{CSA} can predict maximum isometric strength of knee extension in COPD patients and ultrasound with bioimpedance analysis can provide valuable measure of sarcopenia in this patient population.
Maynard-Paquette et al. 2020 [35]	Canada/ O	M=20 F= 0	66±6	Stable	Gold 1,2,3,4	Rectus Femoris	Diagnostic	To evaluate the relationship between quadriceps size, and symptoms, lung function and diaphragm contractility in COPD patients using ultrasound.	➢ Mean Qcsa, Qthick and Qci were 336±145 mm2, 1.55±0.53 cm and 64±16%, respectively, and mean TFdi was 91±36%<br>➢ Qci was significantly correlated with FFMI (rho=0.59, p=0.001), TFdi (rho=0.41, p=0.008), FEV1 (rho=0.43, p=0.001) but not with age (rho=0.18, p=0.28).<br>➢ Qci was significantly correlated to CAT score (rho=−0.47, p=0.002), even when controlled for FEV1, and was lower in patients with an mMRC score ≥2 (55±15 vs 70±14%, p=0.002).<br>➢ Q_{CSA} and Q_{ci} were significantly lower in patients with regular exacerbations<br>➢ Only FFMI and TFdi were found to be significantly related to lower Qci values.<br>➢ Ultrasound evaluation of the quadriceps contractile index is feasible and related to disease severity, clinical symptoms, and exacerbation history and diaphragm contractility.
J M Seymour et al. 2009 [52]	UK/O	COPD M=16 F=14 Control 26	68 (9)	Stable	Gold 1,2,3,4	Rectus Femoris	Diagnostic	To measure the quadriceps cross sectional area in COPD patients using ultrasound method and to relate the measurement to fat-free mass and muscle strength.	➢ There was 25% reduction in mean RF_{CSA} (-115 mm^2; 95% CI=-177 to -54, P=0.001) in COPD compared to healthy controls.<br>➢ RF_{CSA} was related to dyspnoea scale score independent of sex and FFM.<br>➢ Maximum voluntary contraction (MVC) and RF_{CSA} in COPD patients were positively correlated (r = 0.78, p<0.001).<br>➢ Ultrasound method can easily and accurately be used for measuring muscle cross-sectional area in patient with COPD.
Lee KM et al. 2019 [53]	S. Korai/O	COPD M= 126 F= 3<br><br>Control M=139 F=125	71 (65–76)	Stable	Gold 1,2	Endobronchial ultrasound	Diagnostic	To diagnose peripheral lung lesions	➢ The overall diagnostic yield of EBUS in patients with no or mild emphysema was significantly higher than in those with moderate or severe pulmonary emphysema (78% vs. 61%, P = 0.007)<br>➢ EBUS-GS is a safe procedure with an acceptable diagnostic yield
Georgiou et al. 2016 [54]	Australia /O	92 M= F	70.1 ±9.5	Stable	Gold 1-4	Endobronchial ultrasound	Diagnostic	To diagnose peripheral lung lesions	➢ EBUS is a safe and effective procedure for the investigation of peripheral solitary pulmonary nodule in advanced COPD.

Abbreviations: 6MWT: six minute walk test; ADMVC: ankle dorsiflexor maximal voluntary contraction; BALF: bronchioalveolar lavage fluid ; BMD: bone mineral density; BMI: body mass index; COPD: chronic obstructive pulmonary disease; EBUS: Endobronchial ultrasound ; EI: Echo intensity; ET: exercise training; F: female; FACITF: functional assessment of chronic illness therapy fatigue; FEV_1: Forced

expiratory volume in one second; FFM: fat-free mass; FFMI: fat free mass index ; FVC: forced vital capacity; GOLD: global initiative for chronic obstructive lung disease; HRQoL: health-related quality of life ; MMP-9: matrix metallopeptidase 9 ; MVCQ: maximum voluntary contraction for quadriceps; OR: odd ratio; Qci: quadriceps contractile index ; Qcsa: quadriceps cross-sectional area; Qthick: quadriceps thickness; RCT: randomise control trial; SSC: stretch-shortening cycle ; TACSA: Tibialis anterior cross-sectional area ; TA_{CSA}: Tibialis anterior cross-sectional area ; TFdi: Diaphragm thickening fraction ; TGF-β: Transforming growth factor beta; VL: Vastus lateralis

References:

1. Dos Santos Yamaguti WP, Paulin E, Shibao S, et al. Air trapping: The major factor limiting diaphragm mobility in chronic obstructive pulmonary disease patients. *Respirology* 2008;13(1):138-44.
2. Baria MR, Shahgholi L, Sorenson EJ, et al. B-mode ultrasound assessment of diaphragm structure and function in patients with COPD. *Chest* 2014;146(3):680-85.
3. Nair A, Alaparthi GK, Krishnan S, et al. Comparison of Diaphragmatic Stretch Technique and Manual Diaphragm Release Technique on Diaphragmatic Excursion in Chronic Obstructive Pulmonary Disease: A Randomized Crossover Trial. *Pulmonary Medicine* 2019;2019(6364376)
4. Okura K, Kawagoshi A, Iwakura M, et al. Contractile capability of the diaphragm assessed by ultrasonography predicts nocturnal oxygen saturation in COPD. *Respirology*;22(2):301-06.
5. Gorman RB, McKenzie DK, Butler JE, et al. Diaphragm length and neural drive after lung volume reduction surgery. *Am J Respir Crit Care Med* 2005;172(10):1259-66. doi: 10.1164/rccm.200412-1695OC [published Online First: 2005/08/20]
6. Gorman RB, McKenzie DK, Pride NB, et al. Diaphragm length during tidal breathing in patients with chronic obstructive pulmonary disease. *Am J Respir Crit Care Med* 2002;166(11):1461-9. doi: 10.1164/rccm.200111-087OC [published Online First: 2002/10/31]
7. Grosu HB, Ost DE, Lee YI, et al. Diaphragm Muscle Thinning in Subjects Receiving Mechanical Ventilation and Its Effect on Extubation. *Respir Care* 2017;62(7):904-11. doi: 10.4187/respcare.05370 [published Online First: 2017/03/30]
8. El Aziz AAA, Elwahsh RA, Abdelaal GA, et al. Diaphragmatic assessment in COPD patients by different modalities. *Egyptian journal of chest diseases and tuberculosis* 2017;66(2):247-50.
9. Yamaguti WP, Claudino RC, Neto AP, et al. Diaphragmatic breathing training program improves abdominal motion during natural breathing in patients with chronic obstructive pulmonary disease: a randomized controlled trial. *Archives of physical medicine and rehabilitation* 2012;93(4):571-77.
10. Corbellini C, Boussuges A, Villafañe JH, et al. Diaphragmatic mobility loss in subjects with moderate to very severe COPD may improve after in-patient pulmonary rehabilitation. *Respiratory care* 2018;63(10):1271-80.
11. He L, Zhang W, Zhang J, et al. Diaphragmatic motion studied by M-mode ultrasonography in combined pulmonary fibrosis and emphysema. *Lung* 2014;192(4):553-61.
12. Eryüksel E, Cimşit C, Bekir M, et al. Diaphragmatic thickness fraction in subjects at high-risk for COPD exacerbations. *Respiratory care* 2017;62(12):1565-70.
13. Ogan N, Aydemir Y, Evrin T, et al. Diaphragmatic thickness in chronic obstructive lung disease and relationship with clinical severity parameters. *Turkish journal of medical sciences* 2019;49(4):1073-78.
14. Bhatt S, Guleria R, Luqman-Arafath T, et al. Effect of tripod position on objective parameters of respiratory function in stable chronic obstructive pulmonary disease. *The Indian journal of chest diseases & allied sciences* 2009;51(2):83.
15. Evrin T, Korkut S, Ozturk Sonmez L, et al. Evaluating Stable Chronic Obstructive Pulmonary Disease by Ultrasound. *Emergency medicine international* 2019;2019
16. Zhang X, Yuan J, Zhan Y, et al. Evaluation of diaphragm ultrasound in predicting extubation outcome in mechanically ventilated patients with COPD. *Irish Journal of Medical Science (1971-)* 2019:1-8.
17. Sun Q, Liu L, Pan C, et al. Effects of neurally adjusted ventilatory assist on air distribution and dead space in patients with acute exacerbation of chronic obstructive pulmonary disease. *Critical Care* 2017;21(1):126.
18. Priori R, Aliverti A, Albuquerque AL, et al. The effect of posture on asynchronous chest wall movement in COPD. *Journal of Applied Physiology* 2013;114(8):1066-75.
19. Rocha T, Souza H, Brandao DC, et al. The manual diaphragm release technique improves diaphragmatic mobility, inspiratory capacity and exercise capacity in people with chronic

obstructive pulmonary disease: a randomised trial. *Journal of physiotherapy* 2015;61(4):182-89.
20. Elsawy SB. Impact of chronic obstructive pulmonary disease severity on diaphragm muscle thickness. *Egyptian Journal of Chest Diseases and Tuberculosis* 2017;66(4):587-92.
21. Paulin E, Yamaguti W, Chammas M, et al. Influence of diaphragmatic mobility on exercise tolerance and dyspnea in patients with COPD. *Respiratory medicine* 2007;101(10):2113-18.
22. Kang HW, Kim TO, Lee BR, et al. Influence of diaphragmatic mobility on hypercapnia in patients with chronic obstructive pulmonary disease. *Journal of Korean medical science* 2011;26(9):1209-13.
23. Souza RM, Cardim AB, Maia TO, et al. Inspiratory muscle strength, diaphragmatic mobility, and body composition in chronic obstructive pulmonary disease. *Physiotherapy Research International* 2019;24(2):e1766.
24. Lim SY, Lim G, Lee YJ, et al. Ultrasound Assessment Of Diaphragmatic Function During Acute Exacerbation Of Chronic Obstructive Pulmonary Disease: A Pilot Study. *International Journal of Chronic Obstructive Pulmonary Disease* 2019;14:2479.
25. Abbas A, Embarak S, Walaa M, et al. Role of diaphragmatic rapid shallow breathing index in predicting weaning outcome in patients with acute exacerbation of COPD. *International Journal of COPD* 2018;13:1655-61. doi: 10.2147/COPD.S161691
26. McKenzie DK, Gorman RB, Tolman J, et al. Estimation of diaphragm length in patients with severe chronic obstructive pulmonary disease. *Respiration physiology* 2000;123(3):225-34.
27. Jain S, Nair G, Nuchin A, et al. Study of the diaphragm in chronic obstructive pulmonary disease using ultrasonography. *Lung India: Official Organ of Indian Chest Society* 2019;36(4):299.
28. Smargiassi A, Inchingolo R, Tagliaboschi L, et al. Ultrasonographic assessment of the diaphragm in chronic obstructive pulmonary disease patients: relationships with pulmonary function and the influence of body composition-a pilot study. *Respiration* 2014;87(5):364-71.
29. Cimsit C, Bekir M, Karakurt S, et al. Ultrasound assessment of diaphragm thickness in COPD. 2016
30. Marchioni A, Castaniere I, Tonelli R, et al. Ultrasound-assessed diaphragmatic impairment is a predictor of outcomes in patients with acute exacerbation of chronic obstructive pulmonary disease undergoing noninvasive ventilation. *Critical Care* 2018;22(1):109.
31. Crimi C, Heffler E, Augelletti T, et al. Utility of ultrasound assessment of diaphragmatic function before and after pulmonary rehabilitation in COPD patients. *International Journal of Chronic Obstructive Pulmonary Disease* 2018;13:3131.
32. Bhatt SP, Luqman-Arafath T, Gupta AK, et al. Volitional pursed lips breathing in patients with stable chronic obstructive pulmonary disease improves exercise capacity. *Chronic respiratory disease* 2013;10(1):5-10.
33. Andrews SM, Deoghare HV, Mills PK, et al. Pulmonary Rehabilitation Maintenance Program May Prevent Accelerated FEV1 Decline in Patients With COPD. *Clinical Pulmonary Medicine* 2017;24(4):143-48.
34. Scheibe N, Sosnowski N, Pinkhasik A, et al. Sonographic evaluation of diaphragmatic dysfunction in COPD patients. *International journal of chronic obstructive pulmonary disease* 2015;10:1925.
35. Maynard-Paquette A-C, Poirier C, Chartrand-Lefebvre C, et al. Ultrasound Evaluation of the Quadriceps Muscle Contractile Index in Patients with Stable Chronic Obstructive Pulmonary Disease: Relationships with Clinical Symptoms, Disease Severity and Diaphragm Contractility. *International Journal of Chronic Obstructive Pulmonary Disease* 2020;15:79.
36. Maddocks M, Jones M, Snell T, et al. Ankle dorsiflexor muscle size, composition and force with ageing and chronic obstructive pulmonary disease. *Exp Physiol* 2014;99(8):1078-88. doi: 10.1113/expphysiol.2014.080093 [published Online First: 2014/06/15]
37. Greening NJ, Harvey-Dunstan TC, Chaplin EJ, et al. Bedside assessment of quadriceps muscle by ultrasound after admission for acute exacerbations of chronic respiratory disease. *American journal of respiratory and critical care medicine* 2015;192(7):810-16.

38. Ye X, Wang M, Xiao H. Echo intensity of the rectus femoris in stable COPD patients. *International journal of chronic obstructive pulmonary disease* 2017;12:3007.
39. Alcazar J, Losa-Reyna J, Rodriguez-Lopez C, et al. Effects of concurrent exercise training on muscle dysfunction and systemic oxidative stress in older people with COPD. *Scandinavian journal of medicine & science in sports* 2019;29(10):1591-603.
40. Vrieze A, De Greef M, Wýkstra P, et al. Low bone mineral density in COPD patients related to worse lung function, low weight and decreased fat-free mass. *Osteoporosis International* 2007;18(9):1197-202.
41. Wallbridge P, Parry SM, Das S, et al. Parasternal intercostal muscle ultrasound in chronic obstructive pulmonary disease correlates with spirometric severity. *Scientific reports* 2018;8(1):1-9.
42. Seymour JM, Ward K, Raffique A, et al. Quadriceps and ankle dorsiflexor strength in chronic obstructive pulmonary disease. *Muscle & nerve* 2012;46(4):548-54.
43. Coratella G, Rinaldo N, Schena F. Quadriceps concentric-eccentric force and muscle architecture in COPD patients vs healthy men. *Human movement science* 2018;59:88-95.
44. Kaneko H, Maruyama H, Sato H. Relationship between expiratory activity of the lateral abdominal muscle and exercise tolerance in chronic obstructive pulmonary disease. *Journal of Physical Therapy Science* 2008;20(2):147-51.
45. Górka K, Soja J, Jakieła B, et al. Relationship between the thickness of bronchial wall layers, emphysema score, and markers of remodeling in bronchoalveolar lavage fluid in patients with chronic obstructive pulmonary disease. *Pol Arch Med Wewn* 2016;126(6):402-10.
46. Cruz-Montecinos C, Guajardo-Rojas C, Montt E, et al. Sonographic measurement of the quadriceps muscle in patients with chronic obstructive pulmonary disease: functional and clinical implications. *Journal of Ultrasound in Medicine* 2016;35(11):2405-12.
47. Nijholt W, ter Beek L, Hobbelen JS, et al. The added value of ultrasound muscle measurements in patients with COPD: An exploratory study. *Clinical nutrition ESPEN* 2019;30:152-58.
48. Navarro-Cruz R, Alcazar J, Rodriguez-Lopez C, et al. The effect of the stretch-shortening cycle in the force-velocity relationship and its association with physical function in older adults with COPD. *Frontiers in physiology* 2019;10:316.
49. Shrikrishna D, Patel M, Tanner RJ, et al. Quadriceps wasting and physical inactivity in patients with COPD. *European Respiratory Journal* 2012;40(5):1115-22.
50. Menon MK, Houchen L, Harrison S, et al. Ultrasound assessment of lower limb muscle mass in response to resistance training in COPD. *Respiratory research* 2012;13(1):119.
51. Ramírez-Fuentes C, Mínguez-Blasco P, Ostiz F, et al. Ultrasound assessment of rectus femoris muscle in rehabilitation patients with chronic obstructive pulmonary disease screened for sarcopenia: correlation of muscle size with quadriceps strength and fat-free mass. *European Geriatric Medicine* 2019;10(1):89-97.
52. Seymour JM, Ward K, Sidhu PS, et al. Ultrasound measurement of rectus femoris cross-sectional area and the relationship with quadriceps strength in COPD. *Thorax* 2009;64(5):418-23.
53. Lee KM, Lee G, Kim A, et al. Clinical outcomes of radial probe endobronchial ultrasound using a guide sheath for diagnosis of peripheral lung lesions in patients with pulmonary emphysema. *Respiratory research* 2019;20(1):177.
54. Georgiou HD, Taverner J, Irving LB, et al. Safety and efficacy of radial EBUS for the investigation of peripheral pulmonary lesions in patients with advanced COPD. *J Bronchology Interv Pulmonol* 2016;23(3):192-98.
